# Novel mitochondrial gene rearrangements pattern in the millipede *Polydesmus* sp. GZCS‐2019 and phylogenetic analysis of the Myriapoda

**DOI:** 10.1002/ece3.8764

**Published:** 2022-03-24

**Authors:** Qing Zuo, Zhisheng Zhang, Yanjun Shen

**Affiliations:** ^1^ 26463 Key Laboratory of Eco‐Environments in Three Gorges Reservoir Region (Ministry of Education) School of Life Sciences Southwest University Chongqing China; ^2^ 12524 Chongqing Key Laboratory of Animal Biology School of Life Sciences Chongqing Normal University Chongqing China

**Keywords:** evolutionary history, gene rearrangement, Myriapoda

## Abstract

The subphylum Myriapoda included four extant classes (Chilopoda, Symphyla, Diplopoda, and Pauropoda). Due to the limitation of taxon sampling, the phylogenetic relationships within Myriapoda remained contentious, especially for Diplopoda. Herein, we determined the complete mitochondrial genome of *Polydesmus* sp. GZCS‐2019 (Myriapoda: Polydesmida) and the mitochondrial genomes are circular molecules of 15,036 bp, with all genes encoded on + strand. The A+T content is 66.1%, making the chain asymmetric, and exhibits negative AT‐skew (−0.236). Several genes rearrangements were detected and we propose a new rearrangement model: “TD (N\R) L + C” based on the genome‐scale duplication + (non‐random/random) loss + recombination. Phylogenetic analyses demonstrated that Chilopoda and Symphyla both were monophyletic group, whereas Pauropoda was embedded in Diplopoda to form the Dignatha. Divergence time showed the first split of Myriapoda occurred between the Chilopoda and other classes (Wenlock period of Silurian). We combine phylogenetic analysis, divergence time, and gene arrangement to yield valuable insights into the evolutionary history and classification relationship of Myriapoda and these results support a monophyletic Progoneata and the relationship (Chilopoda + (Symphyla + (Diplopoda + Pauropoda))) within myriapod. Our results help to better explain the gene rearrangement events of the invertebrate mitogenome and lay the foundation for further phylogenetic study of Myriapoda.

## Graphical abstract

We determined the complete mitochondrial genome of *Polydesmus* sp. GZCS‐2019 and all genes encoded on + strand. Several genes rearrangements were detected and we propose a new rearrangement model: “TD (N\R) L + C” based on the genome‐scale duplication + (non‐random/random) loss + recombination. We combine phylogenetic analysis, divergence time, and gene arrangement to yield valuable insights into the evolutionary history and classification relationship of Myriapoda and these results support a monophyletic Progoneata and the relationship (Chilopoda + (Symphyla + (Diplopoda + Pauropoda))) within myriapod.

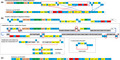

## 1 INTRODUCTION

Now Myriapoda is looked as a subphylum of Arthropoda, including four classes: Pauropoda, Symphyla, Diplopoda (millipedes), Chilopoda (centipedes). It is known that Myriapoda first settled in terrestrial ecosystems in the Early Paleozoic, (Lozano‐Fernandez et al., 2016), with primitive body shapes, making them play a particularly important role in evolutionary analysis (Dunham, 2012; Giribet & Edgecombe, 2019). Morphological studies hypothesized that the Diplopoda and Pauropoda clustered together to form the Dignatha with the second maxillary segment being limbless in the two pairs of gnathal appendages (Dohle, 1980; Pocock, 1893; Shelley & Golovatch, 2011; Tiegs, 1947). In addition, morphology supported that the Symphyla and Dignatha (Pauropoda+Diplopoda) together formed the taxon Progoneata (Dohle, 1980; Pocock, 1893) based on their common morphological characteristics: the location of the genital opening is near the front of the trunk (Blanke & Wesener, 2014). Chilopoda was presumed to have a sister relationship with the Progoneata (Blanke & Wesener, 2014; Dohle, 1980; Edgecombe, 2006, 2011; Gai et al., 2008; Moritz & Brown, 1987; Read & Enghoff, 2009; Wilson & Anderson, 2004).

In recent decades, with the development of molecular biology, a relatively new field of molecular analysis based on mitochondrial and transcriptome datais flourishing. In contrast to this traditional morphology view, several molecular studies contradicted the Dignatha clade and supported Symphyla + Pauropoda group formed Edafopoda (Andreas et al., 2012; Dong et al., 2012; Fernandez et al., 2018; Gai et al., 2006; Rehm et al., 2014). Therefore, the relationship among the four classes of myriapod is still controversial and the major source of conflict is between molecular and morphological phylogeny.

The millipedes (Diplopoda) is an important component of the modern terrestrial ecosystem due to its important role in the decomposition of organic matter. Hitherto, the Diplopoda contained more than 18,000 species worldwide, which are distributed in most parts of China (Golovatch & Liu, 2020; Jiang & Chen, 2018). Although Diplopoda is the third most diverse class of Myriapoda, there is no widely accepted consensus about the classification and phylogenetic relationship. With the development of molecular biology technology, a new era of phylogenetic analysis of phylogeny has been opened in the early 1990s, and a large number of analyses of millipedes have been published (Brewer et al., 2013; Dong et al., 2016; Lavrov et al., 2002; Liu et al., 2017; Means et al., 2021; Qu et al., 2020; Wesener et al., 2010; Zhao et al., 2020). However, due to the limitation of taxon sampling and the lack of mitochondrial genome data, the previous phylogenetic studies failed to solve the relationship of millipedes.

The mitochondrial genomes of metazoans exhibit variation in many characteristics, such as length, tRNA secondary structure, gene rearrangement, and structure of control regions (Boore, 1999; Mukundan et al., 2020; Shen et al., 2017, 2020). Studying the variation in these characteristics can discover the evolutionary relationship between taxa with a high and/or low classification level. Among them, gene arrangements are relatively complex and diverse, which can become a source of information for system evolution analysis. Furthermore, it also affects the process of mRNA transcription, substitution, and processing. In recent years, the mitochondrial genome rearrangement has been widely studied focusing on phylogenetic relationship and rearrangement mechanism (Feng et al., 2021; Gong et al., 2020; Li et al., 2019, 2020; Powell et al., 2020; Tyagi et al., 2020; Wang et al., 2020; Zhang et al., 2020). In addition, the high rearrangement rate makes the Myriapoda an ideal group to study the interaction between gene rearrangement and phylogenetic relationship. For example, the studies discussed the gene arrangement of Myriapoda on phylogenetic inference but did not prove the universality of this mechanism in the same order species (Gai et al., 2008; Lavrov et al., 2002). Some studies found that the gene arrangement pattern was a sound molecular evidence supporting the Helminthomorpha clade (Brewer et al., 2013; Dong et al., 2012), but they did not elaborate the evolutionary implications of gene arrangements in the Myriapoda. Several common models have been used to explain the gene rearrangement events in the current animal mtDNA, for example: recombination models involved in DNA strand breaks and recombination (Lunt & Hyman, 1997); the Tandem duplication‐random loss (TDRL) model is commonly used to support gene tandem replication and random loss (Moritz & Brown, 1987); and the TDNL model supports gene tandem replication and non‐random loss (Lavrov et al., 2002). However, the gene rearrangement phenomenon may not be explained by one of the above‐mentioned mechanisms alone for some species. Therefore, it is necessary to conduct comparative evolutionary studies on mitogenome rearrangements to accurately identify the mechanisms leading to the rearrangements.

In the present study, we sequenced the complete mitochondrial genome of a millipede, *Polydesmus* sp. GZCS‐2019 (*P*. GZCS‐2019) (Diplopoda: Polydesmidae), and described the genome‐scale gene rearrangement events of the mitogenome, providing independent molecular evidence to explore the phylogenetic relationship of Myriapoda. To build a better phylogenetic relationship and understand the evolutionary significance of gene arrangement in Myriapoda, the other 27 complete mitochondrial genomes of the Myriapoda (8 from Chilopoda, 13 from Diplopoda, 2 from Symphyla, and 1 from Pauropoda) and 3 outgroup species were used in this study. Meanwhile, we combine phylogenetic analysis, divergence time, and gene arrangement to yield valuable insights into the evolutionary history and classification relationship of Myriapoda.

## 2 MATERIALS AND METHODS

### 2.1 Specimen collection and DNA extraction

Two specimens of *P*. GZCS‐2019 were collected from Chishui of Guizhou Province in China (28°24′25″N, 105°57′17″E) in August 2019. Morphological identification of specimens was mainly referred to as “PICTORIAL KEYS TO SOIL ANIMALS OF China” (Yin, 1998) and all specimens were stored in anhydrous ethanol in the Chongqing Key Laboratory of Animal Biology, Chongqing Normal University. Total DNA was extracted from the dehydrated muscle tissues using the TaKaRa MiniBEST Universal Genomic DNA Extraction Kit Ver.5.0 (TaKaRa Biotech).

### 2.2 Mitochondrial genome sequencing and assembly

The entire mitogenome of *P*. GZCS‐2019 was sequenced on the Illumina HiSeq TM platform with paired ends of 300–500 bp. The raw paired reads were quality trimmed using FastQC v0.11.4 (www.bioinformatics.babraham.ac.uk/projects/fastqc/) with default parameters. Finally, yielded 10G raw reads (coverage 3–5×) and clean sequence reads were assembled in the NOVOPlasty (https://github.com/ndierckx/NOVOPlasty) (Nicolas et al., 2016) using sequences from each of the 23 mitochondrial genes of the closest relative available from NCBI as mapping reference, with the default parameter.

### 2.3 Sequence analysis and gene annotation

The online tool MITOS (http://mitos2.bioinf.uni‐leipzig.de/index.py) was used to perform gene annotation, and the annotation results were verified by the BLAST program from the NCBI website (https://blast.ncbi.nlm.nih.gov/Blast.cgi) (Donath et al., 2013). Then, the abnormal start codon and stop codon were determined based on the comparison with other millipedes. The relative synonymous codon usage (RSCU) was obtained using MEGA 7.0 (Kumar et al., 2015), which was calculated using PCG with incomplete codons removed. The ribosomal RNA genes were determined according to the location of adjacent tRNA genes and comparison with other Myriapoda mitogenomes from NCBI. The strand asymmetry was calculated using the following formula: AT‐skew = (A − T)/(a + T); GC‐skew = (G − C)/(G + C) (Perna & Kocher, 1995). The online mitochondrial visualization tool Organellar Genome DRAW (Marc et al., 2013) was used to draw a graphical map of the mitochondrial genome. The secondary cloverleaf structure and the locations of tRNAs were examined with tRNAscan‐SE 1.21 (Lowe & Chan, 2016).

### 2.4 Phylogenetic reconstruction

The mitochondrial genomes used for phylogenetic analysis in this study were all from GenBank, including 24 species of Myriapoda and 3 species of outgroup (1 Decapoda species and 2 Hexapoda species). The species information is shown in Table 1. This phylogenetic analysis is based on 37 genes, including 13 protein coding genes (PCG), 2 ribosomal RNA genes (rRNAs) and 22 transfer RNA genes (tRNAs). The sequences above were aligned by ClustalW method in MEGA 7 (Kumar et al., 2015), with the default parameters. The Gblocks version 0.91b (Castresana, 2000) with the default parameters setting was used for filtering of poorly aligned regions. The aligned sequences of each gene were concatenated using Sequence Matrix v1.7 (Castresana, 2000).

**TABLE 1:** Summary of mitogenomic sequence information used in the present study

Species	Taxonomic position	Size (bp)	GenBank no.	Reference
***Polydesmus* sp. GZCS‐2019**	Diplopoda; Helminthomorpha; Polydesmida; Polydesmidae	**15,036**	**MZ677220**	**This study**
*Appalachioria falcifera*	Diplopoda; Helminthomorpha; Polydesmida; Xystodesmidae	15,282	JX437063	Brewer et al. (2013)
*Xystodesmus* sp. YD‐2016	Diplopoda; Helminthomorpha; Polydesmida; Xystodesmidae	15,791	KU721886	Dong et al. (2016)
*Asiomorpha coarctata*	Diplopoda; Helminthomorpha; Polydesmida; Paradoxosomatidae	15,644	KU721885	Dong et al. (2016)
*Anaulaciulus koreanus*	Diplopoda; Helminthomorpha; Julida; Julidae	14,916	KX096886	Unpublished
*Antrokoreana gracilipes*	Diplopoda; Helminthomorpha; Julida; Nemasomatidae	14,747	DQ344025	Unpublished
*Brachycybe lecontii*	Diplopoda; Helminthomorpha; Playtdesmida; Andrognathidae	15,115	JX437064	Brewer et al. (2013)
*Abacion magnum*	Diplopoda; Helminthomorpha; Callipodida; Callipodidae	15,160	JX437062	Brewer et al. (2013)
*Thyropygus* sp. DVL‐2001	Diplopoda; Helminthomorpha; Spirostreptida; Harpagophoridae	15,133	AY055728	Lavrov et al. (2002)
*Narceus annularus*	Diplopoda; Helminthomorpha; Spirobolida; Spirobolidae	14,868	AY055727	Lavrov et al. (2002)
*Sphaerotheriidae* sp. HYS‐2012	Diplopoda; Helminthomorpha; Sphaerotheriida; Sphaerotheriidae	14,970^a^	JQ713564	Dong et al. (2012)
*Glomeridesmus* sp. ITV8918	Diplopoda; Pentazonia; Glomeridesmida; Glomeridesmidae	14,848	MG905160	Unpublished
*Glomeridesmus spelaeus*	Diplopoda; Pentazonia; Glomeridesmida; Glomeridesmidae	14,819	MG372113	Nunes et al. (2020)
*Mecistocephalus marmoratus*	Chilopoda; Pleurostigmophora; Geophilomorpha; Mecistocephalidae	15,279	KX774322	Unpublished
*Strigamia maritima*	Chilopoda; Pleurostigmophora; Geophilomorpha; Linotaeniidae	14,983	KP173664	Robertson et al. (2015)
*Bothropolys* sp. SP‐2004	Chilopoda; Pleurostigmophora; Lithobiomorpha; Ethopolyidae	15,139	AY691655	Unpublished
*Cermatobius longicornis*	Chilopoda; Pleurostigmophora; Lithobiomorpha; Henicopidae	16,833	KC155628	Gai et al. (2013)
*Lithobius forficatus*	Chilopoda; Pleurostigmophora; Lithobiomorpha; Lithobiidae	15,695	AF309492	Lavrov et al. (2000)
*Scolopocryptops* sp. 1 YG‐2013	Chilopoda; Pleurostigmophora; Scolopendromorpha; Cryptopidae	15,119	KC200076	Gai et al. (2014)
*Scolopendra subspinipes dehaani*	Chilopoda; Pleurostigmophora; Scolopendromorpha; Scolopendridae	14,538^a^	KY947341	Unpublished
*Scutigera coleoptrata*	Chilopoda; Notostigmophora; Scutigeromorpha; Scutigeridae	14,922	AJ507061	Negrisolo et al. (2004)
*Symphylella* sp. YG‐2006	Symphyla; Scolopendrellidae	14,667	EF576853	Gai et al. (2008)
*Scutigerella causeyae*	Symphyla; Scutigerellidae	14,637	DQ666065	Podsiadlowski et al. (2007)
*Pauropus longiramus*	Pauropoda; Pauropodidae	14,487	HQ457012	Dong et al. (2012)
Outgroup				
*Japyx solifugus*	Hexapoda; Japygidae	15,785	NC007214	Carapelli et al. (2005)
*Penaeus monodon*	Crustacea; Decapoda; Dendrobranchiata; Penaeidae	15,984	AF217843	Wilson et al. (2000)
*Petrobius brevistylis*	Hexapoda; Machilidae	15,698	NC007688	Podsiadlowski (2006)

Note

Bolded text represents the species in this study.

^a^ Incomplete mitogenome.

Phylogenetic trees were constructed using the following three datasets: (1) 13 PCGs matrices consisting of 9140 nt; (2) 13 PCG and 2 rRNA matrices composed of 10,884 nt; (3) 13 PCGs, 2 rRNAs, and 10 tRNA matrices composed of 11,459 nt. For these three datasets, the best fitting model GTR + I + G was selected by jModelTest 2 (Darriba et al., 2012) for maximum likelihood (ML) and Bayesian inference (BI) analysis. The ML analysis was assembled in PhyML 3.0 (Stéphane & Olivier, 2003) with fast likelihood‐based method and performed 1000 repetitions. Bayesian analyses were carried out using MrBayes 3.1.2 (Ronquist & Huelsenbeck, 2003) under the best‐fit models with 10,000,000 generations in two runs of eight chains each and each one was sampled every 200 generations with a burn‐in of 25%. Trees inferred prior to stationarity were discarded as burn‐in, and those remaining were used to construct a 50% majority rule consensus tree. All phylogenetic trees were viewed and edited using Figtree v1.3.1 (http://tree.bio.ed.ac.uk/software/figtree).

### 2.5 Divergence time estimation

Beast v1.8.4 (Drummond et al., 2012) was used to estimate the divergence time, using the Bayesian analysis method. At the same time, Beauti v1.8.3 was used to generate the beast XML file using uncorrelated lognormal distribution relaxed clock model and the Yule speciation was used to prior process the tree. Two fossil constraints were used in this study: the oldest uncontested terrestrial animal *Pneumodesmus newmani* (421–426 Mya) (Shear et al., 1998) and the oldest terrestrial myriapod body fossil *Rhyniella praecursor* (407–411 Mya) (Wilson & Anderson, 2004). The GTR + I + G model was used to estimate time, and after a burn‐in of the initial 50% cycles, divergence times were sampled once every 1000 generations from 100 million Markov Chain Monte Carlo (MCMC) iterations.

The treeAnnotator v1.6.1 (BEAST software) was used to annotate the sampled trees, and the Figtree v1.3.1 was used to conduct the visualization. The ESSs were used to determining the Bayesian statistical significance of each parameter in TRACER v1.5 (ESS > 200) (Rambaut & Drummond, 2003).

## 3 RESULTS AND DISCUSSION

### 3.1 Genome structure, organization, and composition

**FIGURE 1:**
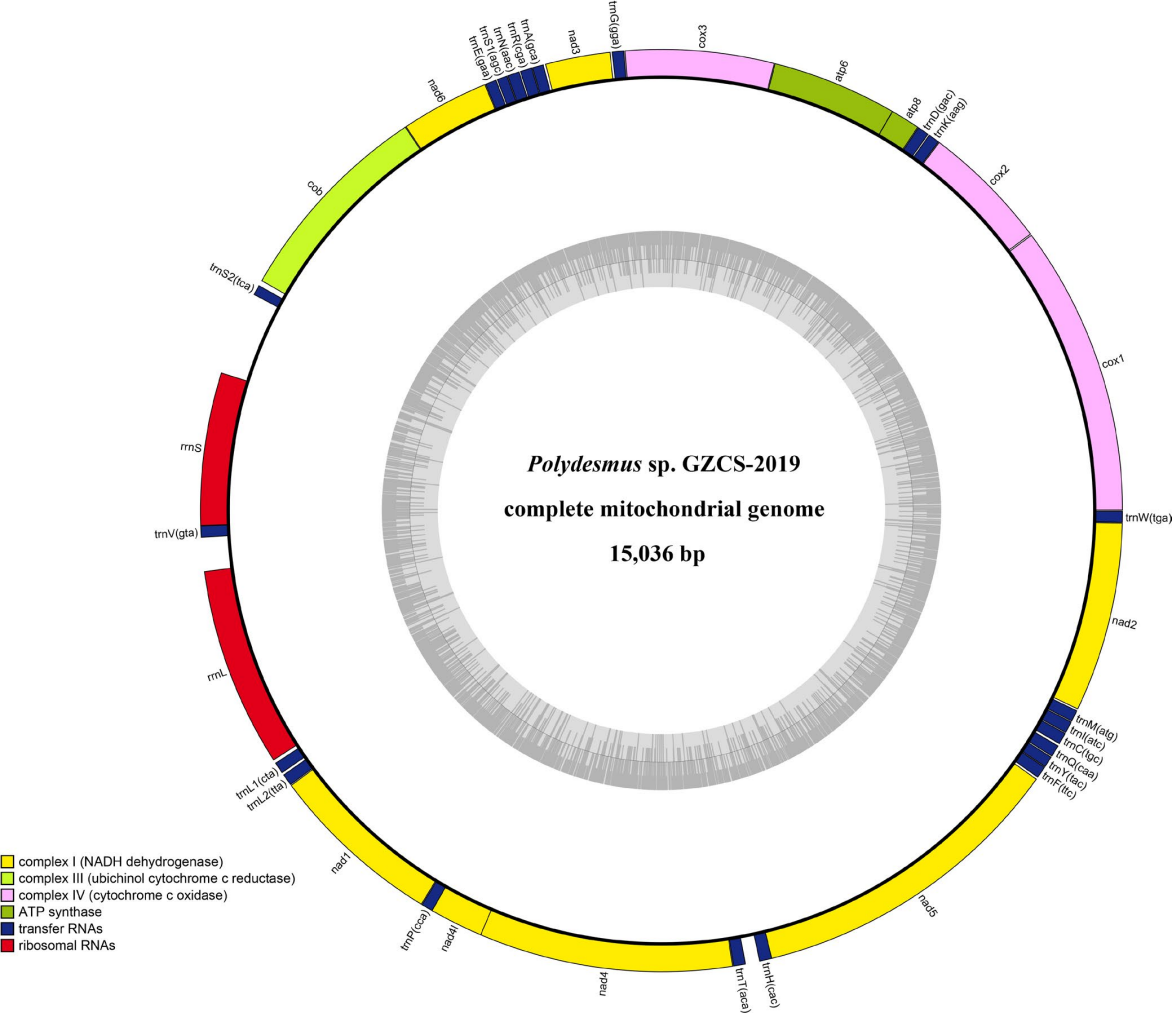
Gene map of the mitochondrial genome *Polydesmus* sp. GZCS‐2019 visualization ring diagram

The complete mitogenome sequence of *P*. GZCS‐2019 is a closed circular molecule with a size of 15,036 bp (Figure 1 and Table 1). In addition, the gene content also conforms to the typical characteristics of other Diplopoda species, including 13 PCGs (*cox1*‐*3*, *nad1*‐*6*, *nad4L*, *cob*, *atp6*, and *atp8*), 2 rRNA genes (*rrnS* and *rrnL*), 22 tRNA genes, and a control region, and all genes are encoded on the heavy (+) chain (Figure 1 and Table 2). Moreover, the mitogenome contains 351 bp intergenic spacer sequences, distributed in 19 regions, ranging in size from 1 to 174 bp (Table 2), and there is a 27 bp overlap between genes in five locations, showing five pairs of overlapping genes: *atp8/atp6*, *rrnS/trnV*, *trnP/nad4L*, *nad4L/nad4*, *and trnH/nad5*, of which the longest 7 bp overlap is located between *trnL1* and *rrnL*, *nad4L* and *nad4*. Furthermore, the whole mitogenome of *P*. GZCS‐2019 is biased toward AT nucleotides (66.1%), similarly to *Abacion magnum* (66.6%), which belongs to the Callipodida (Table 1). The mitogenome of *P*. GZCS‐2019 has been deposited in NCBI under GenBank accession number MZ677220.

**TABLE 2:** Features of the mitochondrial genome of *Polydesmus* sp. GZCS‐2019

Gene	Position no.	Length (bp)	Start codon	Stop codon	Anticodon	Intergenic length	Strand
*cox1*	1–1533	1533	ATG	TAA			+
*cox2*	1537–2214	678	ATG	TAA		+3	+
*trnK*	2215–2278	64			AAG		+
*trnD*	2282–2346	65			GAC	+3	+
*atp8*	2347–2505	159	ATG	TAG			+
*atp6*	2502–3165	664	ATA	T		−4	+
*cox3*	3166–3951	786	ATG	TAA			+
*trnG*	3953–4017	65			GGA	+1	+
*nad3*	4025–4369	345	ATG	TAG		+7	+
*trnA*	4378–4439	62			GCA	+8	+
*trnR*	4440–4505	66			CGA		+
*trnN*	4509–4574	66			AAC	+3	+
*trnS1*	4575–4632	58			AGC		+
*trnE*	4636–4697	62			GAA	+3	+
*nad6*	4698–5166	469	ATG	T			+
*cob*	5167–6271	1105	ATG	T			+
*trnS2*	6297–6353	57			TCA	+25	+
Control region	6354–6790	437					+
*rrnS*	6791–7598	808					+
*trnV*	7596–7659	64			GTA	−3	+
*rrnL*	7834–8887	1054				+174	+
*trnL1*	8903–8966	64			CTA	+15	+
*trnL2*	8977–9042	66			TTA	+10	+
*nad1*	9044–9968	925/952	ATG	T/		+1	+
*trnP*	9969–10,033	65			CCA		+
*nad4L*	10,028–10,315	288	ATC	TAG		−6	+
*nad4*	10,309–11,650	1342	ATG	T		−7	+
*trnT*	11,651–11,717	67			ACA		+
*trnH*	11,789–11,857	69			CAC	+71	+
*nad5*	11,851–13,550	1700	ATG	TA		−7	+
*trnF*	13,559–13,625	67			TTC	+8	+
*trnY*	13,626–13,688	63			TAC		+
*trnQ*	13,691–13,756	66			CAA	+2	+
*trnC*	13,766–13,831	66			TGC	+9	+
*trnI*	13,833–13,898	66			ATC	+1	+
*trnM*	13,900–13,963	64			ATG	+1	+
*nad2*	13,970–14,972	1003	GTG	T		+6	+
*trnW*	14,973–15,035	63			TGA		+

### 3.2 PCGS and codon usage

In the mitogenome of *P*. GZCS‐2019, the size of the PCGs region is 9882 bp, and all the PCGs genes are encoded on the + strand (Table 2). Furthermore, its mitochondrial DNA is similar to that of other invertebrates, with the eight typical PCGs (*nad1*, *cox1*, *cox2*, *cob*, *cox3*, *atp8*, *nad4*, and *nad6*) start with the standard ATG starting codon, *nad2* with GTG as the starting codon, *nad4L* and *nad5* with ATC as it, *atp6* and *nad3* with ATA as it.

Meanwhile, *cox1*, *cox2*, and *cox3* use TAA as the termination codon; *atp8*, *nad3*, and *nad4L* use TAG as the termination codon; *nad5* use TA as the termination codon, while *atp6*, *nad6*, *cob*, *nad4L*, *nad4*, and *nad2* are terminated by a single T (Table 2). These features are somewhat similar to other invertebrate mitochondrial genomes, and the truncated stop codon may be completed in the form of TAA and TAG through post‐transcriptional polyadenylation (Ojala et al., 1981). In the 13 PCGs of *P*. GZCS‐2019, 5012 codons are showed and the most common amino acids are Leu (UUR) (477), Ile (AUR) (295), and Phe (UUR) (462) (Table S1 and Figure S4).

### 3.3 Skewness, transfer RNAs, and ribosomal RNAs

The nucleotide composition of the mitogenome of *P*. GZCS‐2019 is as follow: A (25.3%), T (40.9%), G (24.2%), and C (9.7%) (Table 3). The whole mitochondrial genome of *P*. GZCS‐2019 exhibits chain asymmetry. The AT‐skew of this whole mitochondrial genome is negative (−0.236), indicating that the occurrence of Ts is higher than that of As. At the same time, the GC‐skew of this whole mitochondrial genome is positive (+0.429), indicating that the occurrence of Gs is higher than that of Cs. Similar results were observed in *Asiomorpha coarctata*, *Xystodesmus* sp. YD‐2016, and *Appalachioria falcifera*. Ultimately, the nucleotide bias was assessed (Table 3), and millipede from the same order gave similar results, the negative AT‐skew and positive GC‐skew is a common feature of Polydesmida.

**TABLE 3:** Composition and skewness of *Polydesmus* sp. GZCS‐2019 mitogenomes in this study

*Polydesmus* sp. GZCS−2019	Size (bp)	A%	T%	G%	C%	AT (%)	GC (%)	AT‐skew	GC‐skew
Mitogenome	**15,036**	**25.3**	**40.9**	**24.2**	**9.7**	**66.1**	**33.9**	**−0.236**	**0.429**
PCGs	10,997	22.5	42.3	25.1	9.9	64.9	35.1	−0.305	0.430
*cox1*	1533	22.9	42.6	13.4	21.1	65.5	34.5	−0.301	0.225
*cox2*	678	24.0	40.6	22.7	12.7	64.6	35.4	−0.256	0.283
*cox3*	786	19.34	42.2	27.2	11.2	61.6	38.4	−0.372	0.417
*nad1*	925	22.6	42.8	25.4	9.2	65.4	34.5	−0.309	0.469
*nad2*	1003	23.2	44.5	7.7	24.6	67.7	32.3	−0.314	0.525
*nad3*	345	19.4	42.9	6.7	31.0	62.3	37.7	−0.377	0.646
*nad4*	1342	21.4	42.2	27.3	9.2	63.6	36.4	−0.327	0.496
*nad4L*	288	22.2	38.9	32.9	5.9	61.1	38.9	−0.272	0.696
*nad5*	1700	23.6	42.8	25.4	8.2	66.4	33.6	−0.289	0.513
*nad6*	469	24.5	41.8	26.0	7.7	66.3	33.7	−0.260	0.544
*atp6*	664	22.9	42.5	23.5	11.1	65.4	34.6	−0.299	0.356
*atp8*	159	28.3	39.6	27.0	5.0	67.9	32.1	−0.167	0.686
*cob*	1105	21.7	41.7	24.1	12.5	63.4	36.6	−0.315	0.317
tRNAs	1415	31.2	36.6	22.0	10.1	67.8	32.2	−0.079	0.371
rRNAs	1862	33.1	37.1	20.8	8.97	70.2	29.8	−0.056	0.397
Control region	437	34.6	38.4	21.9	5.0	72.9	27.0	−0.053	0.627

Note

Bolded text represents the species in this study.

In the mitochondrial genome of *P*. GZCS‐2019, there are 22 tRNAs encoded on the + strand and with a typical cloverleaf structure, which are the common characteristics of the mitogenome of most millipedes. At the same time, the size of these tRNAs was between 57 and 69 bp, showing a strong A+T bias (67.8%) and a slight skew of T versus A (AT‐skew = −0.079) (Table 3). The canonical cloverleaf secondary structure is observed in all the other tRNAs except *trnS1* and *trnS2* without dihydrouridine (DHU) arm (Figure S2). In general, such deletion of DHU arm in the secondary structure of *trnS1* and *trnS2* was considered a common condition in the Diplopoda mitogenome (Brewer et al., 2013; Dong et al., 2016). The stems of cloverleaf secondary include mostly normal base pairs and multiple non‐Watson–Crick base airs. Furthermore, the most common non‐Watson–Crick base pair is G–U (or U–G) wobble base pairs, which have been known to provide comparable thermodynamic stability to Watson–Crick base pairs and are nearly isomorphic to them. The G–U (or U–G) base pairs appear in all 22 tRNAs.

The *rrnS* (1054 bp) gene located between Control region and *trnV*, and the *rrnL* (808 bp) gene located between *trnV* and *trnL1* are encoded on + strand (Table 1 and Figure 1). The A+T content (70.2%) of the rRNA genes is higher than the whole genome (66.1%) (Table 3), with a negative AT‐skew −0.056 (Table 2) and whose structural diagram is shown in Figure S3.

### 3.4 Control regions

**FIGURE 2:**
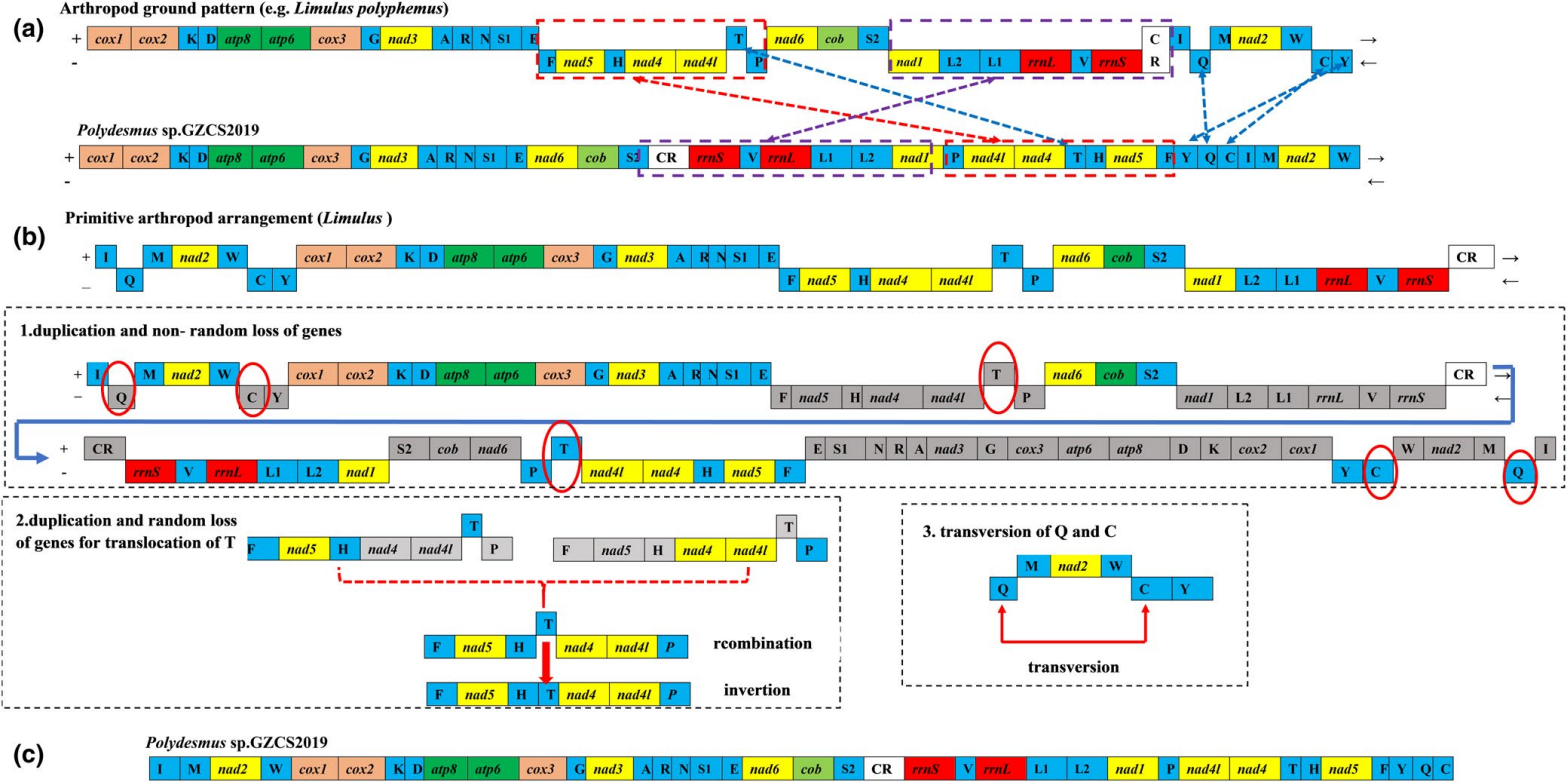
Inferred intermediate steps between the ancestral gene arrangement of myriapod and *Polydesmus* sp. GZCS‐2019 mitogenomes. PCGs, CR, and tRNAs are indicated with boxes. Genes labeled above the diagram are encoded on the + strand and those below the diagram on the − strand. The lost genes are labeled with gray. (a) The ancestral gene arrangement of myriapod. (b(1)) Two monomers derivative from the duplication of ancestor arranged in a circular dimer. Subsequently, non‐random loss is followed according to the orientation of transcription for each gene. (b(2)) Tandem duplication followed by random deletion (TDRL) lead to the translocation of trnT. (b(3)) The recombination model lead to transversion of trnC‐trnQ. (c) This part is the final result of the genetic rearrangement of the *P.* GZCS‐2019 mitogenome

The largest non‐coding region of the mitochondrial genome is usually presumed as the control region and is heavily biased toward A+T nucleotides. The four Polydesmida species mitogenome: *P*. GZCS‐2019, *X*. YD‐2016, *A*. *coarctata*, and *A*. *falcifera* are compared with the ancestor *Limulus polyphemus*. We found the non‐coding regions of Polydesmida vary in number, size, and location due to the duplications and rearrangement of genome (Figure 2). Our analyses suggested that non‐coding region, which is heavily biased to A+T nucleotides, located between *trnS2* and *rrnS* was a putative control region. Besides, there are some common conserved motifs observed in the four Polydesmida species (Figure S7), including the hairpin loop structures, TA(A)n‐like stretch, TATA motif, and G(A)nT motif, which were identified as initiation sites for replication and transcription (Boore, 1999; Cameron et al., 2007; Jeffrey, 1999; Shadel & Clayton, 1997; Taanman, 1999; Wei et al., 2013). However, the poly A‐stretches at the 5′ and 3′ end of the ancestor *L*. *polyphemus* are not observed in the other three Polydesmida species (Figure S6). Therefore, we speculate that this event is responsible for the reverse of strand transcription direction observed in this Polydesmida order and further experiments are needed to clarify this speculation.

### 3.5 Gene rearrangement

Compared with ancestral Arthropoda (e.g., *L*. *polyphemus*), seven genes and gene blocks (*trnF*‐*nad5*‐*trnH*‐*nad4*‐*nad4L*‐*trnT*‐*trnP*, *nad1*‐*trnL2*‐*trnL1*‐*rrnL*‐*trnV*‐*rrnS*, *trnT*, *trnC*, *trnY*, *trnI*, and *trnQ*), and AT‐rich region (putative control region; CR) have been rearranged in *P*. GZCS‐2019 (Figure 2a). The mitogenome of *P*. GZCS‐2019 is unique compared to other myriapod species; all coding regions are on a single strand. At present, several mature mechanisms have been commonly used to explain gene rearrangement in animal mitogenomes, including duplication‐random loss (TDRL) (Moritz & Brown, 1987), duplication‐nonrandom loss (TDNL) (Lavrov et al., 2002), and recombination (Lunt & Hyman, 1997). However, several unique features of *P*. GZCS‐2019 rearrangements prevent the application of these models to this species.

Here, we propose a new rearrangement model: “TD (N\R) L + RC” model based on a genome‐scale duplication + (non‐random/random) loss + recombination account for the mitogenome gene rearrangement of the *P*. GZCS‐2019. In deducing the rearrangement mechanism of this mito genome, with reference to the theory of the non‐random loss (TDNL) model (Lavrov et al., 2002) all but minor rearrangements were found: the *trnT* translocation and the *trnI‐trnQ* translocation. The first step is the tandem duplication of the entire mitogenome, resulting in a dimeric molecule with two identical monomers covalently linked head to tail (Figure 2b1). Consecutive copies were then followed by a non‐random loss of the duplicated genes and the loss of genes would be predetermined by their transcriptional polarity. All genes having one polarity would be lost from one genome copy, and all genes having the opposite polarity would be lost from the other, ending with monomer 1 (***trnI***, *trnQ*, ***trnM***, ***nad2***, ***trnW***, *trnC*, *trnY*, ***cox1***, ***cox2***, ***trnK***, ***trnD***, ***atp8***, ***atp6***, ***cox3***, ***trnG***, ***nad3***, ***trnA***, ***trnR***, ***trnN***, ***trnS1***, ***trnE***, *trnF*, *nad5*, *trnH*, *nad4*, *nad4L*, ***trnT***, *trnP*, ***nad6***, ***cob***, ***trnS2***, *nad4L*, *trnL2*, *trnL1*, *rrnL*, *trnV*, *rrnS*, and ***CR***) and monomer 2 (***CR***, *rrnS*, *trnV*, *rrnL*, *trnL1*, *trnL2*, *nad4L*, ***trnS2***, ***cob***, ***nad6***, *trnP*, ***trnT***, *nad4L*, *nad4*, *trnH*, *nad5*, *trnF*, ***trnE***, ***trnS1***, ***trnN***, ***trnR***, ***trnA***, ***nad3***, ***trnG***, ***cox3***, ***atp6***, ***atp8***, ***trnD***, ***trnK***, ***cox2***, ***cox1***, *trnC*, ***trnW***, ***nad2***, ***trnM***, *trnQ*, and ***trnI***) (underline denotes the deleted gene; the bold ones are genes that are encoded in the + strand; and the regular ones are genes that are encoded in the − strand) (Figure 2b1). Different from the TDNL model (Lavrov et al., 2002), the 3′ end of monomer 1 is linked to the 3′ end of monomer 2 to form the ultimate gene arrangement of the *P*. GZCS‐2019 mitogenome: (***trnI***, ***trnM***, ***nad2***, ***trnW***, ***cox1***, ***cox2***, ***trnK***, ***trnD***, ***atp8***, ***atp6***, ***cox3***, ***trnG***, ***nad3***, ***trnA***, ***trnR***, ***trnN***, ***trnS1***, ***trnE***, ***nad6***, ***cob***, ***trnS2***, ***CR***, *rrnS*, *trnV*, *rrnL*, *trnL1*, *trnL2*, *nad4L*, *trnP*, *nad4L*, *nad4*, *trnT*, *trnH*, *nad5*, *trnF*, *trnY*, *trnQ*, *trnC*) (the bold ones are genes that are encoded in the + strand; the regular ones are genes that are encoded in the − strand) and the transcription polarity of these genes encoding on the negative strand is reversed, which was shown in Figure 2c. It may be that the non‐coding sequences determined and the predicted possible secondary structures play some roles in the early stages of the replication and transcription process (Lavrov et al., 2002; Parker et al., 2009; Tomita et al., 2002). However, further experiments are needed to clarify this speculation.

Tandem duplication‐random loss mechanism was widely used to explain the translocation of mitochondrial genes, the *trnT* translocation phenomenon in this study can be explained by this theory, that occurring in the region between *nad4L* and *trnP*, followed by deletions of redundant genes resulting in *trnT*‐*nad4*‐*nad4L* (Figure 2b2). In contrast, the inversion of *trnC*‐*trnQ* referred to the transversion of *trnI*‐*trnQ*, which was more in line with the recombination model (Lunt & Hyman, 1997) (Figure 2b3).

Different from TDNL and TDRL, the TD (N\R) L + RC model has the following characteristics: the whole genome duplicated and the gene loss according to their transcriptional polarity but not randomly as in the TDRL; the second is the change in transcription direction and polarity around the control region, which is different from the TDNL method. Indeed, each step of the TD(N\R) L+RC model does not violate the nature and rules of mitochondrial replication. Nevertheless, our presumed model still needs more experimental evidence to verify.

We compared the gene order of *P*. GZCS‐2019 mitogenome with another three Polydesmida species (*A*. *coarctata*, *X*. YD‐2016, and *A*. *falcifera*). A striking finding is that all of them were almost arranged in the same way and all coding regions were on a single strand (Figure S5), indicating that this may be a common feature of Polydesmida, and showing that *P*. GZCS‐2019 had a close evolution connection with *A*. *coarctata*, *X*. YD‐2016, and *A. falcifera*. Furthermore, we also found the inversion of the entire side of a genome (*trnF*‐*nad5*‐*trnH*‐*nad4*‐*nad4L*, *trnP*, *nad1*‐*trnL2*‐*trnL1*‐*rrnL*‐*trnV*‐*rrnS*‐*CR*, *trnQ*, *trnC*, and *trnY*) and the translocation of *trnT* and the inversion of *trnC*‐*trnQ* could be proposed as common events about gene order in Polydesmida lineage (Figure S5). The duplication‐nonrandom loss was also detected in the Symphyla species (Gai et al., 2006, 2008), which reinforce the sister relationship with Diplopoda. These results of the regular gene arrangement in Myriapoda provide useful information for the phylogenetic inference of advanced groups.

### 3.6 Phylogenetic reconstruction

**FIGURE 3:**
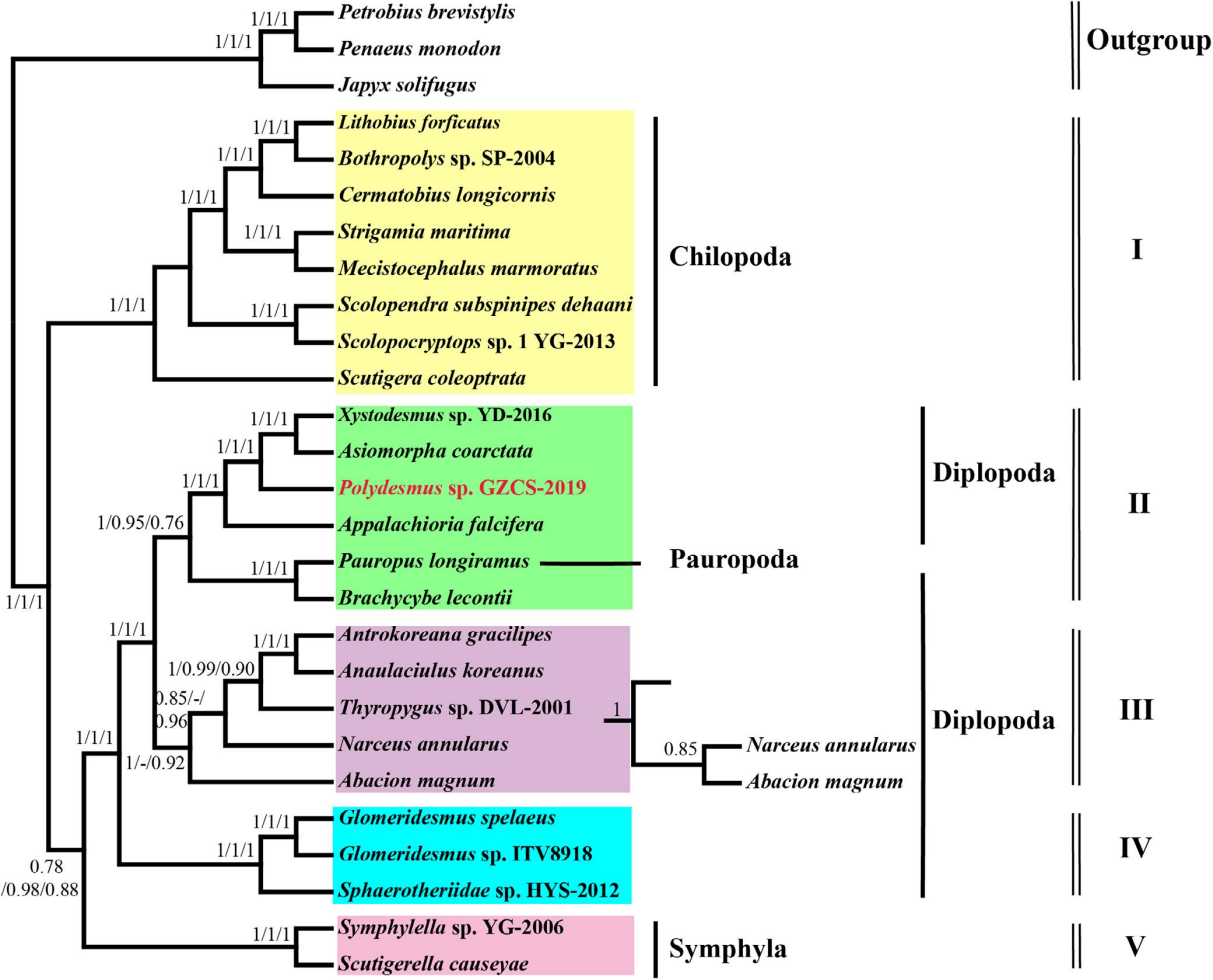
The Phylogenetic tree of *Polydesmus* sp. GZCS‐2019 is based on 13 mitogenome PCGs nucleotide sequences using BI methods. Only Bootstrap support (BP) greater than 50% are shown; the numbers on the branches are bootstrap values for Bayesian posterior probabilities (BPP)

**FIGURE 4:**
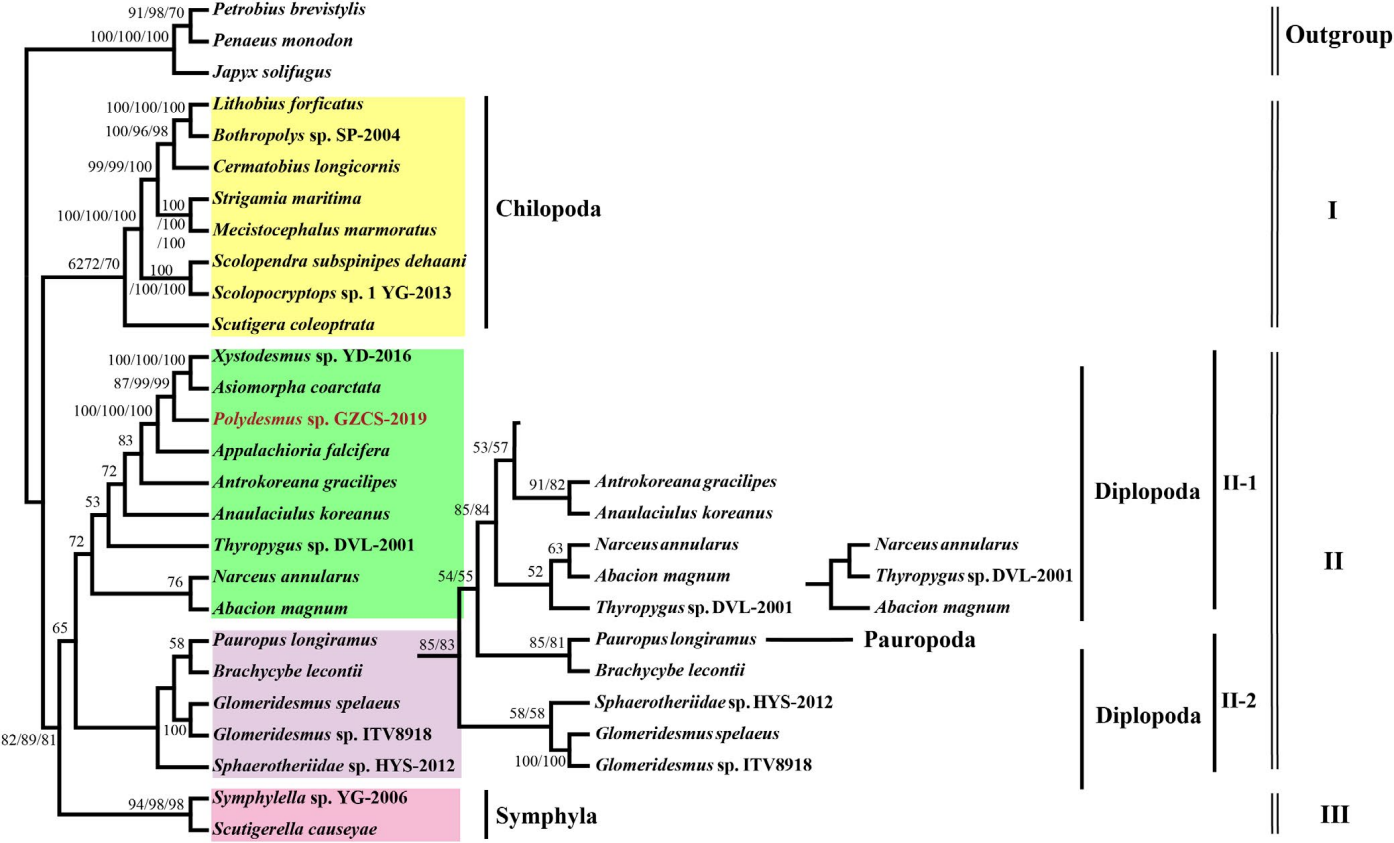
The phylogenetic tree was inferred from the nucleotide sequences of 13 mitogenome PCGs using ML methods. Only Bootstrap support (BP) greater than 50% are shown; the numbers on the branches are bootstrap values for maximum likelihood bootstrapping values

The concatenated set of nucleotide sequences of the 13 PCGs from 13 Diplopoda species, 8 Chilopoda species, 2 Symphyla species, 1 Pauropoda species, and 3 outgroup species are used for reconstructing phylogenetic relationships among the millipedes by BI and ML methods (Figures 3, 4). In this study, conserved blocks of amino acid and nucleotide data sets were used to perform the Bayesian inference and maximum likelihood phylogenetic analysis. Phylogenetic analyses based on three datasets matrix demonstrated the relationships of Myriapoda. Both the BI and ML trees support a sister group relationship of Diplopoda + Pauropoda (named Dignatha), which contradicts the Symphyla + Pauropoda group (named Edafopoda) (Figures 3, 4). The Dignatha group was inferred from morphological data, which shares modified mouthparts, due to the lack of appendage buds on the second maxillary segment (Blanke & Wesener, 2014; Liu et al., 2017; Pocock, 1893). Symphyla is speculated from the BI and ML trees as a sister group of Dignatha (Figures 3, 4), traditional morphology classifies it with Dignatha as Progoneata (Diplopoda + Pauropoda + Symphyla) based on their common morphological characteristics: the location of the genital opening is near the front of the trunk (Dohle, 1980; Edgecombe, 2011; Gai et al., 2008; Pocock, 1893; Sierwald & Bond, 2007; Verhoeff, 1913). Meanwhile, the BI and ML analyses showed the basal position of Chilopoda and the interordinal relationships within the Chilopoda (((Lithobiomorpha + Geophilomorpha) Scolopendromorpha) Scutigeromorpha) (Figures 3, 4, Figure S1B), which was inconsistent with the previous morphological and molecular studies (Bonato et al., 2015; Edgecombe, 2006; Negrisolo et al., 2004).

The Diplopoda was the most numerous species in this study and the extant Diplopoda is divided into two groups: Chilognatha and Penicillata (Blanke & Wesener, 2014; Dohle, 1980; Jiang & Chen, 2018). The Penicillata only includes Polyxenida with no species in our analyses (Figures 3, 4). The Chilognatha includes most of the species in the Diplopoda, which is composed of two monophyletic infraclass Pentazonia and Helminthomorpha (Figure 3, BPP = 1/1/1). The infraclass of Pentazonia is further classified into three orders: Glomerida, Sphaerotheriida, and Glomeridesmida (Figure S1A), contrary to the standard morphological hypothesis that combines Glomerida and Sphaerotheriida into a single clade called Oniscomorpha (Blanke & Wesener, 2014; Iniesta & Wesener, 2012; Sierwald & Bond, 2007). The infraclass Helminthomorpha is composed of two subterclasses: Colobognatha and Eugnatha (Figure S1A). Some previous studies support the monophyly of the Helminthomorpha (Brewer et al., 2013; Dong et al., 2016; Pitz & Sierwald, 2010), however, our phylogenetic trees could not support the monophyly of Helminthomorpha, Colobognatha, and Eugnatha because of the incorporation of the Pauropoda (*Pauropus longiramus*). Nonetheless, the BI analyses in this study strongly supported the sister relationship of Pentazonia and Helminthomorpha (Figure 3 and Figure S1A, BPP = 1/1/1). The morphology studies showed the subterclass Eugnatha is composed of two sister superorders: Juliformia and Polydesmida (Blanke & Wesener, 2014; Jiang & Chen, 2018; Minelli, 2011), which was strongly supported by our results (Figure 3 and Figure S1A, BPP = 1/1/1). Simultaneously, a sister relationship among Julida, Spirostreptida, and Spirobolida was also strongly supported within the superorder Juliformia (Figure 3 and Figure S1A, BPP = 1/1/1), which is consistent with classical taxonomy (Dohle, 1980; Enghoff et al., 1993; Fortey & Thomas, 1998; Pocock, 1893). Additionally, we found that *P*. GZCS‐2019, *A*. *coarctata*, *X*. YD‐2016, and *A*. *falcifera* are clustered in one branch with high support value (Figures 3, 4, Figure S1A, BPP = 1/1/1/, ML = 100/100/100). This phenomenon is also supported by the mitochondrial gene rearrangement model deduced above. Although mitochondrial genome gene rearrangement may provide more phylogenetic markers in this study, the analysis of Myriapoda gene rearrangement pattern cannot fully explain the problem of phylogeny, and may have a certain bias on a single pedigree or branch with low support. In the later stage, we can reconstruct the phylogenetic relationship of Myriapoda more effectively based on the data of the nuclear genome.

### 3.7 Divergence time

**FIGURE 5:**
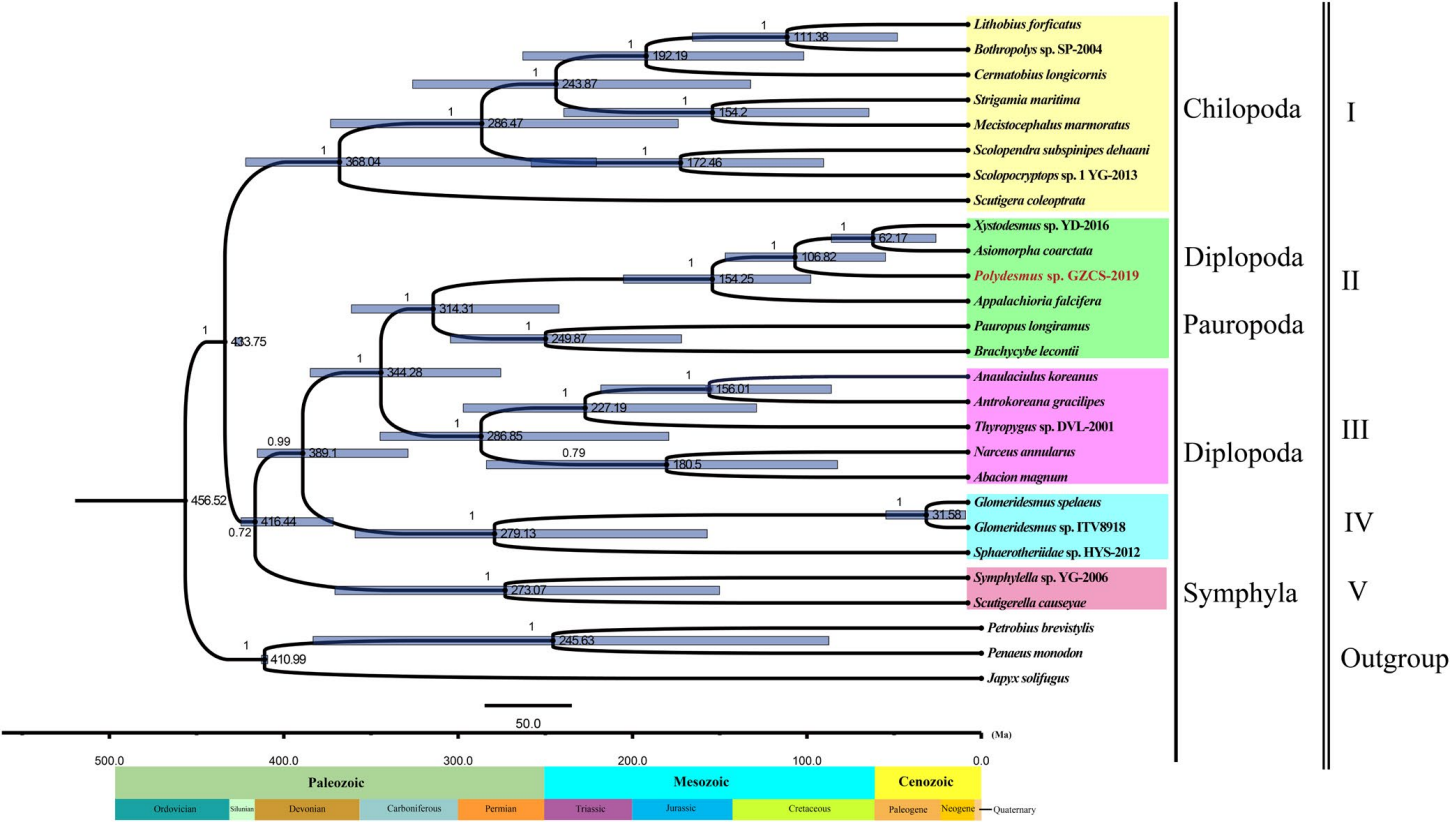
The divergence time estimation of the major myriapod lineages using the Bayesian relaxed molecular clock method in BEAST from two fossil constraint ages based on the best scoring maximum‐likelihood tree. Node bars indicate 95% CIs of the divergence time estimate

Understanding the origin and evolutionary history of myriapods is crucial for interpreting the colonization and evolution of arthropods on land. Hitherto, Myriapoda is inferred to have colonized land in the Early Cambrian, substantially predating body or trace fossil evidence (Giribet & Edgecombe, 2019; Lozano‐Fernandez et al., 2016; Wilson & Anderson, 2004). In this study, the Bayesian divergence times showed that the splitting of the ancestral lineages of the Progoneata and Chilopoda from a common ancestor occurred during the Wenlock period of Silurian, slightly earlier than the oldest millipedes and centipedes fossil records in the Silurian, which was similar to many previous studies (Rosa et al., 2016; Wilson & Anderson, 2004), suggesting that both had experienced the same period of relative stasis period as the plants prior to this (Giribet & Edgecombe, 2019; Lozano‐Fernandez et al., 2016; Minter et al., 2017). Then, the Progoneata clade split into Dignatha (Diplopoda + Pauropoda) and Symphyla during the early Silurian to lower Devonian. The results showed that the first split of myriapod occurred between the subclass Chilopoda and other subclasses, rather than between Symphyla and other subclasses, which align with the morphology classification results that supports the monophyly of Progoneata (Figure 5).

In Chilopoda, the split time of Pleurostigmophora and Notostigmophora was from the early Silurian to middle Triassic, slightly earlier than the oldest fossil chilopods in the Late Silurian (Wilson & Anderson, 2004), which was consistent with some previous studies and these were representatives of the Chilopoda (Bonato et al., 2015; Chipman et al., 2014; Giribet & Ed Gecombe, 2013). Moreover, this study also concluded that the split time of Scolopendromorpha and (Lithobiomorpha + Geophilomorpha) was from middle Devonian to early Jurassic and the divergence time of Lithobiomorpha and Geophilomorpha was from early Carboniferous to early Cretaceous (Figure 5).

In Diplopoda, the divergence time of the two infraclasses, Pentazonia and Helminthomorpha, was in the late Silurian to Pennsylvanian Carboniferous, during the infraclass Helminthomorpha. During the subterclass Eugnatha, the divergence time between the superorders Juliformia and Polydesmida was from early Devonian to middle Permian. Within the infraclass Pentazonia, the divergence time between the superorders Glomeridesmida and Sphaerotheriida was from the late Devonian to the late Jurassic period (Figure 5).

## 4 CONCLUSION

In this paper, we report the complete mitogenome of *P*. GZCS‐2019 (Diplopoda: Polydesmidae) with a novel genome‐scale rearrangement phenomenon. We deduce the genome‐scale “duplication + (non‐random/random) loss + recombination (TD (N\R) L + RC)” model resulted in a novel mechanism of gene rearrangement for the published Polydesmida mitogenome. The deletion of the DHU arm of *trnS1* and *trnS2* was considered a common condition in the Polydesmida mitogenome. The phylogenetic analysis supported the monophyletic of Diplopoda, providing evidence for the higher‐level relationships within it. Meanwhile, we combine phylogenetic analysis and divergence time to yield valuable insights into the evolutionary history and classification relationship of Myriapoda and these results support a monophyletic Progoneata and the relationship (Chilopoda + (Symphyla + (Diplopoda + Pauropoda))).

Since the mitochondrial gene rearrangement events in Myriapoda contain genetic information related to the phylogenetic evolution of species, it is necessary to conduct in‐depth research and use the genetic information revealed by gene rearrangement to better solve these controversial phylogenetic problems. However, due to the lack of taxon samples, there are still many limitations in this study. Therefore, it is necessary to collect more species of Myriapoda for more in‐depth and systematic research.

## CONFLICT OF INTEREST

The authors declare that they have no conflict of interest.

## AUTHOR CONTRIBUTIONS

**Qing Zuo:** Resources (supporting); Writing – original draft (supporting); Writing – review & editing (supporting). **Zhisheng Zhang:** Conceptualization (equal); Data curation (equal); Investigation (equal). **Yanjun Shen:** Formal analysis (equal); Funding acquisition (equal); Investigation (equal).

## Supporting information

Supplementary Material

## Data Availability

DNA sequences: GenBank (Accession Numbers MZ677220).

## References

[ece38764-bib-0001] Andreas, Z. , Regier, J. C. , Zwickl, D. J. , & Gadagkar, S. R. (2012). Resolving discrepancy between nucleotides and amino acids in deep‐level arthropod phylogenomics: Differentiating serine codons in 21‐amino‐acid models. PLoS One, 7, e47450. 23185239 10.1371/journal.pone.0047450 PMC3502419

[ece38764-bib-0002] Bernt, M. , Donath, A. , Jühling, F. , Externbrink, F. , Florentz, C. , Fritzsch, G. , Pütz, J. , Middendorf, M. , & Stadler, P. F. (2013). MITOS: Improved de novo metazoan mitochondrial genome annotation. Molecular Phylogenetics & Evolution, 69, 313–319. 22982435 10.1016/j.ympev.2012.08.023

[ece38764-bib-0003] Blanke, A. , & Wesener, T. (2014). Revival of forgotten characters and modern imaging techniques help to produce a robust phylogeny of the Diplopoda (Arthropoda, Myriapoda). Arthropod Structure & Development, 43, 63–75. 10.1016/j.asd.2013.10.003 24184600

[ece38764-bib-0004] Bonato, L. , Drago, L. , & Murienne, J. (2015). Phylogeny of Geophilomorpha (Chilopoda) inferred from new morphological and molecular evidence. Cladistics – The International Journal of the Willi Hennig Society, 30, 485–507. 10.1111/cla.12060 34794246

[ece38764-bib-0005] Boore, J. L. (1999). Animal mitochondrial genomes. Nucleic Acids Research, 27, 1767–1780. 10.1093/nar/27.8.1767 10101183 PMC148383

[ece38764-bib-0006] Brewer, M. S. , Swafford, L. , Spruill, C. L. , & Bond, J. E. (2013). Arthropod phylogenetics in light of three novel millipede (Myriapoda: Diplopoda) mitochondrial genomes with comments on the appropriateness of mitochondrial genome sequence data for inferring deep level relationships. PLoS One, 8, e68005. 10.1371/journal.pone.0068005 23869209 PMC3712015

[ece38764-bib-0007] Cameron, S. L. , Johnson, K. P. , & Whiting, M. F. (2007). The mitochondrial genome of the screamer louse Bothriometopus (Phthiraptera: Ischnocera): Effects of extensive gene rearrangements on the evolution of the genome. Journal of Molecular Evolution, 65, 589–604. 10.1007/s00239-007-9042-8 17925995

[ece38764-bib-0101] Carapelli, A. , Nardi, F. , Dallai, R. , Boore, J. L. , Liò, P. , & Frati, F. (2005). Relationships between hexapods and crustaceans based on four mitochondrial genes. Crustacean Issues, 16, 295.

[ece38764-bib-0008] Castresana, J. (2000). GBLOCLKS: Selection of conserved blocks from multiple alignments for their use in phylogenetic analysis. Version 0.91b. Molecular Biology & Evolution, 17, 540–552. 10.1093/oxfordjournals.molbev.a026334 10742046

[ece38764-bib-0009] Chipman, A. D. , Ferrier, D. E. K. , Brena, C. , Qu, J. , Hughes, D. S. T. , Schröder, R. , Torres‐Oliva, M. , Znassi, N. , Jiang, H. , Almeida, F. C. , Alonso, C. R. , Apostolou, Z. , Aqrawi, P. , Arthur, W. , Barna, J. C. J. , Blankenburg, K. P. , Brites, D. , Capella‐Gutiérrez, S. , Coyle, M. , … Richards, S. (2014). The first myriapod genome sequence reveals conservative arthropod gene content and genome organisation in the centipede *Strigamia maritima* . PLoS Biology, 12, e1002005. 10.1371/journal.pbio.1002005 25423365 PMC4244043

[ece38764-bib-0010] Darriba, D. , Taboada, G. L. , Doallo, R. , & Posada, D. (2012). jModelTest 2: More models, new heuristics and parallel computing. Nature Methods, 9, 772. 10.1038/nmeth.2109 PMC4594756 22847109

[ece38764-bib-0011] Dohle, W. (1980). Sind die Myriapoden eine monophyletische Gruppe? Eine Diskussion der Verwandtschaftsbeziehungen der Antennaten. Abhandlungen des Naturwissenschaftlichen Vereins in Hamburg, 23, 45–103.

[ece38764-bib-0012] Dong, Y. , Sun, H. , Guo, H. , Pan, D. , Qian, C. , Hao, S. , & Zhou, K. (2012). The complete mitochondrial genome of *Pauropus longiramus* (Myriapoda: Pauropoda): Implications on early diversification of the myriapods revealed from comparative analysis. Gene, 505, 57–65. 10.1016/j.gene.2012.05.049 22659693

[ece38764-bib-0013] Dong, Y. , Zhu, L. , Bai, Y. , Ou, Y. , & Wang, C. (2016). Complete mitochondrial genomes of two flat‐backed millipedes by next‐generation sequencing (Diplopoda, Polydesmida). ZooKeys, 637, 1–20. 10.3897/zookeys.637.9909 PMC5240118 28138271

[ece38764-bib-0014] Drummond, A. J. , Suchard, M. A. , Xie, D. , & Rambaut, A. (2012). Bayesian phylogenetics with BEAUti and the BEAST 1.7. Molecular Biology and Evolution, 22, 1185–1192. 10.1093/molbev/mss075 PMC3408070 22367748

[ece38764-bib-0015] Dunham, J. (2012). Encyclopedia of biodiversity. Library Journal, 137, 100.

[ece38764-bib-0016] Edgecombe, G. G. D. (2006). Conflict between datasets and phylogeny of centipedes: An analysis based on seven genes and morphology. Proceedings Biological Sciences, 273, 531–538. 16537123 10.1098/rspb.2005.3365 PMC1560052

[ece38764-bib-0017] Edgecombe, G. D. (2011). 1 Phylogenetic relationships of Myriapoda. In A. Minelli (Ed.), Treatise on zoology‐anatomy, taxonomy, biology. The Myriapoda (Vol. 1, pp. 1–20). Brill.

[ece38764-bib-0018] Enghoff, H. , Dohle, W. , & Blower, J. G. (1993). Anamorphosis in millipedes (Diplopoda)—the present state of knowledge with some developmental and phylogenetic considerations. Zoological Journal of the Linnean Society, 109, 103–234. 10.1111/j.1096-3642.1993.tb00305.x

[ece38764-bib-0019] Feng, J. , Guo, Y. , Yan, C. , Ye, Y. , Yan, X. , Li, J. , Xu, K. , Guo, B. , & Lü, Z. (2021). Novel gene rearrangement in the mitochondrial genome of *Siliqua minima* (Bivalvia, Adapedonta) and phylogenetic implications for Imparidentia. PLoS One, 16, e0249446. 10.1371/journal.pone.0249446 33822813 PMC8023497

[ece38764-bib-0020] Fernandez, R. , Edgecombe, G. D. , & Giribet, G. (2018). Phylogenomics illuminates the backbone of the Myriapoda tree of life and reconciles morphological and molecular phylogenies. Scientific Reports, 8, 83. 10.1038/s41598-017-18562-w 29311682 PMC5758774

[ece38764-bib-0021] Fortey, R. A. , & Thomas, R. H. (1998). Myriapod‐insect relationships as opposed to an insect‐crustacean sister group relationship (Vol. 55). Springer Netherlands.

[ece38764-bib-0102] Gai, Y. , Ma, H. , Ma, J. , Li, C. , & Yang, Q. (2014). The complete mitochondrial genome of Scolopocryptops sp. (Chilopoda: Scolopendromorpha: Scolopocryptopidae). Mitochondrial DNA, 25(3), 192–193. 23631366 10.3109/19401736.2013.792073

[ece38764-bib-0103] Gai, Y. , Ma, H. , Sun, X. , Ma, J. , Li, C. , & Yang, Q. (2013). The complete mitochondrial genome of Cermatobius longicornis (Chilopoda: Lithobiomorpha: Henicopidae). Mitochondrial DNA, 24(4), 331–332. 23351039 10.3109/19401736.2012.760078

[ece38764-bib-0022] Gai, Y. H. , Song, D. X. , Sun, H. Y. , & Zhou, K. Y. (2006). Myriapod monophyly and relationships among myriapod classes based on nearly complete 28S and 18S rDNA sequences. Zoolog, 23, 1101–1108. 10.2108/zsj.23.1101 17261924

[ece38764-bib-0023] Gai, Y. , Yang, X. Q. , Song, D. D. , Sun, H. , & Zhou, K. (2008). The complete mitochondrial genome of *Symphylella* sp (Myriapoda: Symphyla): Extensive gene order rearrangement and evidence in favor of Progoneata. Molecular Phylogenetics & Evolution, 49, 574–585. 10.1016/j.ympev.2008.08.010 18782622

[ece38764-bib-0024] Giribet, G. , & Edgecombe, G. D. (2013). Stable phylogenetic patterns in scutigeromorph centipedes (Myriapoda: Chilopoda: Scutigeromorpha): Dating the diversification of an ancient lineage of terrestrial arthropods. Invertebrate Systematics, 27(5), 485–501.

[ece38764-bib-0025] Giribet, G. , & Edgecombe, G. D. (2019). The phylogeny and evolutionary history of arthropods. Current Biology, 29, R592–R602. 10.1016/j.cub.2019.04.057 31211983

[ece38764-bib-0026] Golovatch, S. I. , & Liu, W. (2020). Diversity, distribution patterns, and fauno‐genesis of the millipedes (Diplopoda) of mainland China. ZooKeys, 930, 153–198. 10.3897/zookeys.930.47513 32390752 PMC7200884

[ece38764-bib-0027] Gong, L. , Lu, X. , Luo, H. , Zhang, Y. , Shi, W. , Liu, L. , Lü, Z. , Liu, B. , & Jiang, L. (2020). Novel gene rearrangement pattern in *Cynoglossus melampetalus* mitochondrial genome: New gene order in genus *Cynoglossus* (Pleuronectiformes: Cynoglossidae). International Journal of Biological Macromolecules, 149, 1232–1240. 10.1016/j.ijbiomac.2020.02.017 32032708

[ece38764-bib-0028] Iniesta, L. F. M. , Ferreira, R. L. , & Wesener, T. (2012). The first troglobitic Glomeridesmus from Brazil, and a template for a modern taxonomic description of Glomeridesmida (Diplopoda). Zootaxa, 3550, 26–42. 10.11646/zootaxa.3550.1.2

[ece38764-bib-0029] Jeffrey, L. (1999). Survey and summary animal mitochondrial genomes. Nucleic Acids Research, 27, 1767–1780. 10.1093/nar/27.8.1767 10101183 PMC148383

[ece38764-bib-0030] Jiang, X. , & Chen, H. (2018). Research progress in taxonomy of diplopoda in China. Guizhou Science, 5, 56–61.

[ece38764-bib-0031] Kumar, S. , Stecher, G. , & Tamura, K. (2015). MEGA7: Molecular evolutionary genetics analysis version 7.0 for bigger datasets. Molecular Biology and Evolution, 33, 1870–1874. 10.1093/molbev/msw054 PMC8210823 27004904

[ece38764-bib-0104] Lavrov, D. V. , Brown, W. M. , & Boore, J. L. (2000). A novel type of RNA editing occurs in the mitochondrial tRNAs of the centipede Lithobius forficatus. Proceedings of the National Academy of Sciences, 97(25), 13738–13742. 10.1073/pnas.250402997 PMC17645 11095730

[ece38764-bib-0032] Lavrov, D. V. , Boore, J. L. , & Brown, W. M. (2002). Complete mtDNA sequences of two millipedes suggest a new model for mitochondrial gene rearrangements: Duplication and nonrandom loss. Molecular Biology and Evolution, 19, 163–169. 10.1093/oxfordjournals.molbev.a004068 11801744

[ece38764-bib-0033] Li, Q. , Ren, Y. , Shi, X. , Peng, L. , Zhao, J. , Song, Y. , & Zhao, G. (2019). Comparative mitochondrial genome analysis of two ectomycorrhizal fungi (Rhizopogon) reveals dynamic changes of intron and phylogenetic relationships of the subphylum Agaricomycotina. International Journal of Molecular Sciences, 20, 5167. 10.3390/ijms20205167 PMC6829451 31635252

[ece38764-bib-0034] Li, R. , Zhang, W. , Ma, Z. X. , & Zhou, C. F. (2020). Novel gene rearrangement pattern in the mitochondrial genomes of *Torleya mikhaili* and *Cincticostella fusca* (Ephemeroptera: Ephemerellidae). International Journal of Biological Macromolecules, 165, 3106–3114. 10.1016/j.ijbiomac.2020.10.124 33098898

[ece38764-bib-0035] Liu, W. , Wesener, T. , Golovatch, S. , & Tian, M. (2017). Contributions to the millipede genus *Nepalella* Shear, 1979 from China, with four new species and first results on phylogeny based on DNA‐barcoding (Diplopoda, Chordeumatida, Megalotylidae). Zootaxa, 4243, 455. 10.11646/zootaxa.4243.3.3 28610139

[ece38764-bib-0036] Lowe, T. M. , & Chan, P. P. (2016). tRNAscan‐SE on‐line: Integrating search and context for analysis of transfer RNA genes. Nucleic Acids Research, 44, W54–W57. 27174935 10.1093/nar/gkw413 PMC4987944

[ece38764-bib-0037] Lozano‐Fernandez, J. , Carton, R. , Tanner, A. R. , Puttick, M. N. , Blaxter, M. , Vinther, J. , Olesen, J. , Giribet, G. , Edgecombe, G. D. , & Pisani, D. (2016). A molecular palaeobiological exploration of arthropod terrestrialization. Philosophical Transactions of the Royal Society B: Biological Sciences, 371, 20150133. 10.1098/rstb.2015.0133 PMC4920334 27325830

[ece38764-bib-0038] Lunt, D. H. , & Hyman, B. C. (1997). Animal mitochondrial DNA recombination. Nature, 387, 247. 10.1038/387247a0 9153388

[ece38764-bib-0039] Marc, L. , Oliver, D. , Sabine, K. , & Ralph, B. (2013). OrganellarGenomeDRAW—A suite of tools for generating physical maps of plastid and mitochondrial genomes and visualizing expression data sets. Nucleic Acids Research, 41, W575–W581. 23609545 10.1093/nar/gkt289 PMC3692101

[ece38764-bib-0040] Means, J. C. , Hennen, D. A. , Tsutomu, T. , & Marek, P. E. (2021). Phylogenetic systematics of the millipede family Xystodesmidae. Insect Systematics and Diversity, 5, 1–26. 10.1093/isd/ixab003

[ece38764-bib-0041] Minelli, A. (2011). Treatise on zoology – Anatomy, taxonomy, biology. The Myriapoda (Vol. 1, pp. 230–268). Brill. 10.1163/9789004188266

[ece38764-bib-0042] Moritz, C. , & Brown, W. M. (1987). Tandem duplications in animal mitochondrial DNAs: Variation in incidence and gene content among lizards. Proceedings of the National Academy of Sciences of the United States of America, 84, 7183–7187. 10.1073/pnas.84.20.7183 3478691 PMC299254

[ece38764-bib-0043] Mukundan, L. P. , Sukumaran, S. , Sebastian, W. , & Gopalakrishnan, A. (2020). Characterization of the whole mitogenome of largehead hairtail *Trichiurus lepturus* (Trichiuridae): Insights into special characteristics. Biochemical Genetics, 58, 430–451. 10.1007/s10528-020-09956-z 32170439

[ece38764-bib-0045] Minter, N. J. , Buatois, L. A. , Mángano, M. G. , Davies, N. S. , Gibling, M. R. , MacNaughton, R. B. , & Labandeira, C. C. (2017). Early bursts of diversification defined the faunal colonization of land. Nature Ecology & Evolution, 1(7), 1–10.28812620

[ece38764-bib-0044] Negrisolo, E. , Minelli, A. , & Valle, G. (2004). Extensive gene order rearrangement in the mitochondrial genome of the centipede *Scutigera coleoptrata* . Journal of Molecular Evolution, 58, 413–423. 10.1007/s00239-003-2563-x 15114420

[ece38764-bib-0046] Nicolas, D. , Patrick, M. , & Guillaume, S. (2016). NOVOPlasty: De novo assembly of organelle genomes from whole genome data. Nucleic Acids Research, 45, e18. 10.1093/nar/gkw955 PMC5389512 28204566

[ece38764-bib-0105] Nunes, G. L. , Oliveira, R. R. M. , Pires, E. S. , Pietrobon, T. , & Vasconcelos, S. (2020). Complete mitochondrial genome of glomeridesmus spelaeus (diplopoda, glomeridesmida), a troglobitic species from iron‐ore caves in eastern amazon. Mitochondrial DNA Part B, 5(3), 3290–3291. 10.1080/23802359.2020.1812450 PMC7782276 33458136

[ece38764-bib-0047] Ojala, D. , Montoya, J. , & Attardi, G. (1981). tRNA punctuation model of RNA processing in human mitochondria. Nature, 290, 470–474. 10.1038/290470a0 7219536

[ece38764-bib-0048] Parker, S. , Hansen, L. , Abaan, H. O. , Tullius, T. D. , & Margulies, E. H. (2009). Local DNA topography correlates with functional noncoding regions of the human genome. Science, 324, 389–392. 10.1126/science.1169050 19286520 PMC2749491

[ece38764-bib-0049] Perna, N. T. , & Kocher, T. D. (1995). Patterns of nucleotide composition at fourfold degenerate sites of animal mitochondrial genomes. Journal of Molecular Evolution, 41, 353–358. 10.1007/BF01215182 7563121

[ece38764-bib-0050] Pitz, K. M. , & Sierwald, P. (2010). Phylogeny of the millipede order Spirobolida (Arthropoda: Diplopoda: Helminthomorpha). Cladistics, 26, 497–525. 10.1111/j.1096-0031.2009.00303.x 34875768

[ece38764-bib-0106] Podsiadlowski, L. (2006). The mitochondrial genome of the bristletail Petrobius brevistylis (Archaeognatha: Machilidae). Insect Molecular Biology, 15(3), 253–258. 16756544 10.1111/j.1365-2583.2006.00640.x

[ece38764-bib-0107] Podsiadlowski, L. , Kohlhagen, H. , & Koch, M. (2007). The complete mitochondrial genome of Scutigerella causeyae (Myriapoda: Symphyla) and the phylogenetic position of Symphyla. Molecular Phylogenetics and Evolution, 45(1), 251–260. 17764978 10.1016/j.ympev.2007.07.017

[ece38764-bib-0051] Pocock, R. I. (1893). On the classification of the Tracheate Arthropoda—A correction. Nature, 49, 124. 10.1038/049124b0

[ece38764-bib-0052] Powell, C. , Caleca, V. , Rhode, C. , da Costa, L. T. , & van Asch, B. (2020). New mitochondrial gene rearrangement in *Psyttalia concolor*, *P. humilis* and *P. lounsburyi* (Hymenoptera: Braconidae), three parasitoid species of economic interest. Insects, 11, 854. 10.3390/insects11120854 PMC7761351 33276418

[ece38764-bib-0053] Qu, Z. , Nong, W. , So, W. L. , Barton‐Owen, T. , Li, Y. , Leung, T. C. N. , Li, C. , Baril, T. , Wong, A. Y. P. , Swale, T. , Chan, T.‐F. , Hayward, A. , Ngai, S.‐M. , & Hui, J. H. L. (2020). Millipede genomes reveal unique adaptations during myriapod evolution. PLoS Biology, 18, e3000636. 10.1371/journal.pbio.3000636 32991578 PMC7523956

[ece38764-bib-0054] Rambaut, A. , & Drummond, A. J. (2003). Tracer: MCMC trace analysis tool. University of Oxford.

[ece38764-bib-0055] Read, H. J. , & Enghoff, H. (2009). The order Siphonophorida – A taxonomist's nightmare? Lessons from a Brazilian collection. Soil Organisms, 81, 543–556.

[ece38764-bib-0056] Rehm, P. , Meusemann, K. , Borner, J. , Misof, B. , & Burmester, T. (2014). Phylogenetic position of Myriapoda revealed by 454 transcriptome sequencing. Molecular Phylogenetics and Evolution, 77, 25–33. 10.1016/j.ympev.2014.04.007 24732681

[ece38764-bib-0108] Robertson, H. E. , Lapraz, F. , Rhodes, A. C. , & Telford, M. J. (2015). The complete mitochondrial genome of the geophilomorph centipede Strigamia maritima. PLoS One, 10(3), e0121369. 10.1371/journal.pone.0121369 25794168 PMC4368715

[ece38764-bib-0057] Ronquist, F. , & Huelsenbeck, J. P. (2003). MrBayes 3: Bayesian phylogenetic inference under mixed models. Bioinformatics, 19, 1572–1574. 10.1093/bioinformatics/btg180 12912839

[ece38764-bib-0058] Rosa, F. , Edgecombe, G. D. , & Gonzalo, G. (2016). Exploring phylogenetic relationships within Myriapoda and the effects of matrix composition and occupancy on phylogenomic reconstruction. Systematic Biology, 65, 871–889. 27162151 10.1093/sysbio/syw041 PMC4997009

[ece38764-bib-0059] Shadel, G. S. , & Clayton, D. A. (1997). Mitochondrial DNA maintenance in vertebrates. Annual Review of Biochemistry, 66, 409–435. 10.1146/annurev.biochem.66.1.409 9242913

[ece38764-bib-0060] Shear, W. A. , Jeram, A. J. , & Selden, P. (1998). Centiped legs (Arthropoda, Chilopoda, Scutigeromorpha) from the Silurian and Devonian of Britain and the Devonian of North America. American Museum Novitates, 3231, 1–16.

[ece38764-bib-0061] Shelley, R. M. , & Golovatch, S. I. (2011). Atlas of myriapod biogeography. I. Indigenous ordinal and supra‐ordinal distributions in the Diplopoda: Perspectives on taxon origins and ages, and a hypothesis on the origin and early evolution of the class. Center for Systematic Entomology, 158, 1–134.

[ece38764-bib-0062] Shen, Y. , Kou, Q. , Zhong, Z. , Li, X. , He, L. , He, S. , & Gan, X. (2017). The first complete mitogenome of the South China deep‐sea giant isopod *Bathynomus* sp (Crustacea: Isopoda: Cirolanidae) allows insights into the early mitogenomic evolution of isopods. Ecology and Evolution, 7, 1869–1881. 28331594 10.1002/ece3.2737 PMC5355201

[ece38764-bib-0063] Shen, Y. , Yang, N. , Liu, Z. , Chen, Q. , & Li, Y. (2020). Phylogenetic perspective on the relationships and evolutionary history of the Acipenseriformes. Genomics, 112, 3511–3517. 10.1016/j.ygeno.2020.02.017 32105795

[ece38764-bib-0064] Sierwald, P. , & Bond, J. E. (2007). Current status of the myriapod class Diplopoda (Millipedes): Taxonomic diversity and phylogeny. Annual Review of Entomology, 52, 401–420. 10.1146/annurev.ento.52.111805.090210 17163800

[ece38764-bib-0065] Stéphane, G. , & Olivier, G. (2003). A simple, fast, and accurate algorithm to estimate large phylogenies by maximum likelihood. Systematic Biology, 52, 696–704. 10.1080/10635150390235520 14530136

[ece38764-bib-0066] Taanman, J. W. (1999). The mitochondrial genome: Structure, transcription, translation and replication. Biochimica et Biophysica Acta, 1410, 103–123. 10.1016/S0005-2728(98)00161-3 10076021

[ece38764-bib-0067] Tiegs, O. W. (1947). The development and affinities of the Pauropoda, based on a study of *Pauropus silvaticus* . Journal of Cell Science, 88, 275–336. 20253180

[ece38764-bib-0068] Tomita, K. , Yokobori, S. I. , Oshima, T. , Ueda, T. , & Watanabe, K. (2002). The Cephalopod Loligo bleekeri mitochondrial genome: Multiplied noncoding regions and transposition of tRNA genes. Journal of Molecular Evolution, 54, 486–500. 10.1007/s00239-001-0039-4 11956687

[ece38764-bib-0069] Tyagi, K. , Chakraborty, R. , Cameron, S. L. , Sweet, A. D. , Chandra, K. , & Kumar, V. (2020). Rearrangement and evolution of mitochondrial genomes in Thysanoptera (Insecta). Scientific Reports, 10, 695. 10.1038/s41598-020-57705-4 31959910 PMC6971079

[ece38764-bib-0070] Verhoeff, K. W. (1913). Die Ordnungen der Proterandria und zur Kenntnis der Cambaliden (ber Diplopoden. 65. Aufsatz.). Zoologischer Anzeiger, 43, 49–65.

[ece38764-bib-0072] Wang, C. , Chen, H. , Tian, S. , Yang, C. , & Chen, X. (2020). Novel gene rearrangement and the complete mitochondrial genome of *Cynoglossus monopus*: Insights into the envolution of the family Cynoglossidae (Pleuronectiformes). International Journal of Molecular Sciences, 21, 6895. 10.3390/ijms21186895 PMC7555148 32962212

[ece38764-bib-0073] Wei, S. , Dong, X. L. , Wang, Z. M. , Miao, X. G. , & Kong, X. Y. (2013). Complete mitogenome sequences of four flatfishes (Pleuronectiformes) reveal a novel gene arrangement of L‐strand coding genes. BMC Evolutionary Biology, 13, 173. 23962312 10.1186/1471-2148-13-173 PMC3751894

[ece38764-bib-0074] Wesener, T. , Raupach, M. J. , & Sierwald, P. (2010). The origins of the giant pill‐millipedes from Madagascar (Diplopoda: Sphaerotheriida: Arthrosphaeridae). Molecular Phylogenetics and Evolution, 57, 1184–1193. 10.1016/j.ympev.2010.08.023 20813191

[ece38764-bib-0075] Wilson, H. M. , & Anderson, L. I. (2004). Morphology and taxonomy of paleozoic millipedes (Diplopoda: Chilognatha: Archipolypoda) form Scotland. Journal of Paleontology, 78, 169–184.

[ece38764-bib-0109] Wilson, K. , Cahill, V. , Ballment, E. , & Benzie, J. (2000). The complete sequence of the mitochondrial genome of the crustacean Penaeus monodon: Are malacostracan crustaceans more closely related to insects than to branchiopods? Molecular Biology and Evolution, 17(6), 863–874. 10833192 10.1093/oxfordjournals.molbev.a026366

[ece38764-bib-0071] Yin, W. (1998). Pictorical keys to soil animals of China. Science press.

[ece38764-bib-0076] Zhang, Y. , Gong, L. I. , Lu, X. , Jiang, L. , Liu, B. , Liu, L. , Lü, Z. , Li, P. , & Zhang, X. U. (2020). Gene rearrangements in the mitochondrial genome of *Chiromantes eulimene* (Brachyura: Sesarmidae) and phylogenetic implications for Brachyura. International Journal of Biological Macromolecules, 162, 704–714. 10.1016/j.ijbiomac.2020.06.196 32590088

[ece38764-bib-0077] Zhao, Y. , Yu, J. , & Liu, W. (2020). A molecular‐based phylogeny of the millipede genus *Sphaerobelum* Verhoeff, 1924, with the first record of the genus from mainland China (Diplopoda: Sphaerotheriida: Zephroniidae). Annales Societe Entomologique de France, 56, 1–8.

