# Supplementary material for: Novel mitochondrial gene rearrangements pattern in the millipede Polydesmus sp. GZCS‐2019 and phylogenetic analysis of the Myriapoda
Source: Ecol Evol. 2022 Mar 24;12(3):e8764. doi: 10.1002/ece3.8764 (PMC8948135; doi:10.1002/ece3.8764)
Supplement: Supplementary file 1 — Supplementary Material [file ECE3-12-e8764-s001.docx]

**Supplementary Materias**


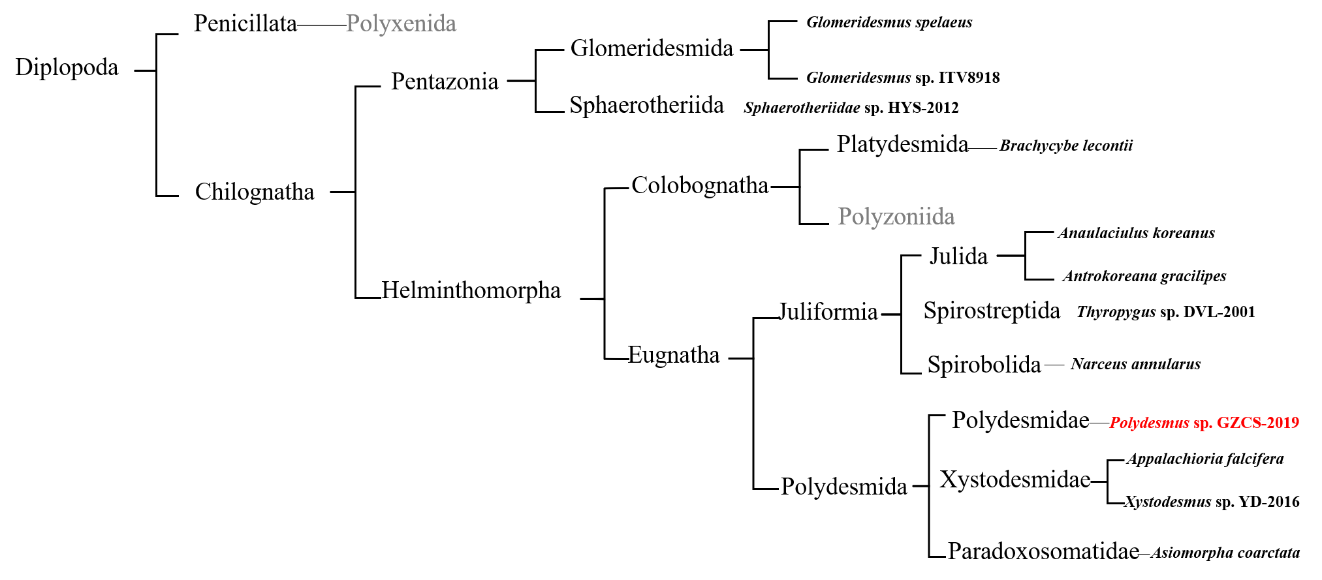


A


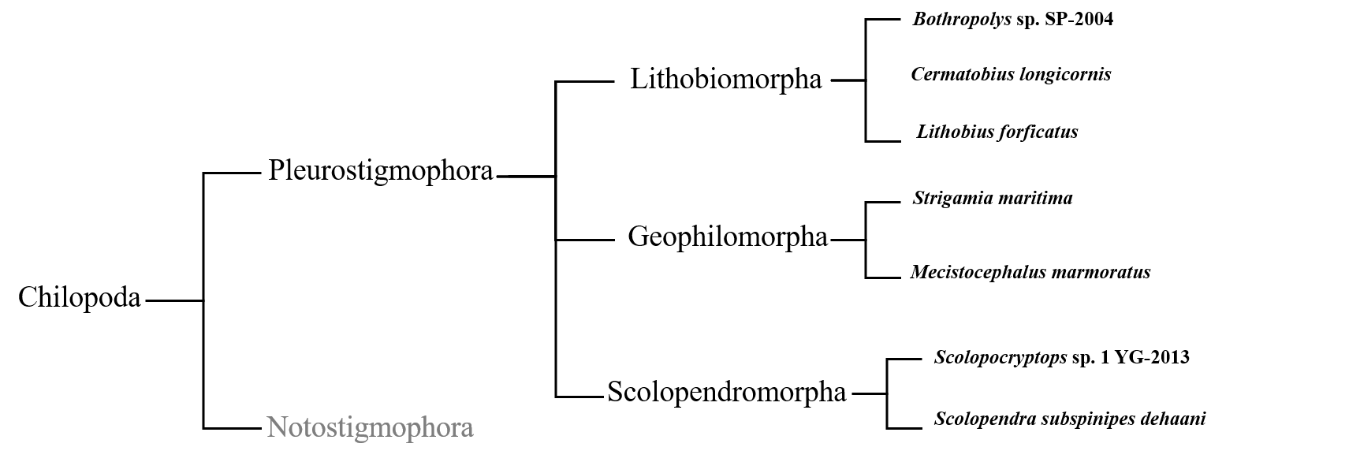


B

**Figure S1.** Schematic summary of the relationship between classes and orders in (A) Diplopoda and (B) Chilopoda based on mitogenome phylogenomics analysis. Species not included in this study were marked gray.


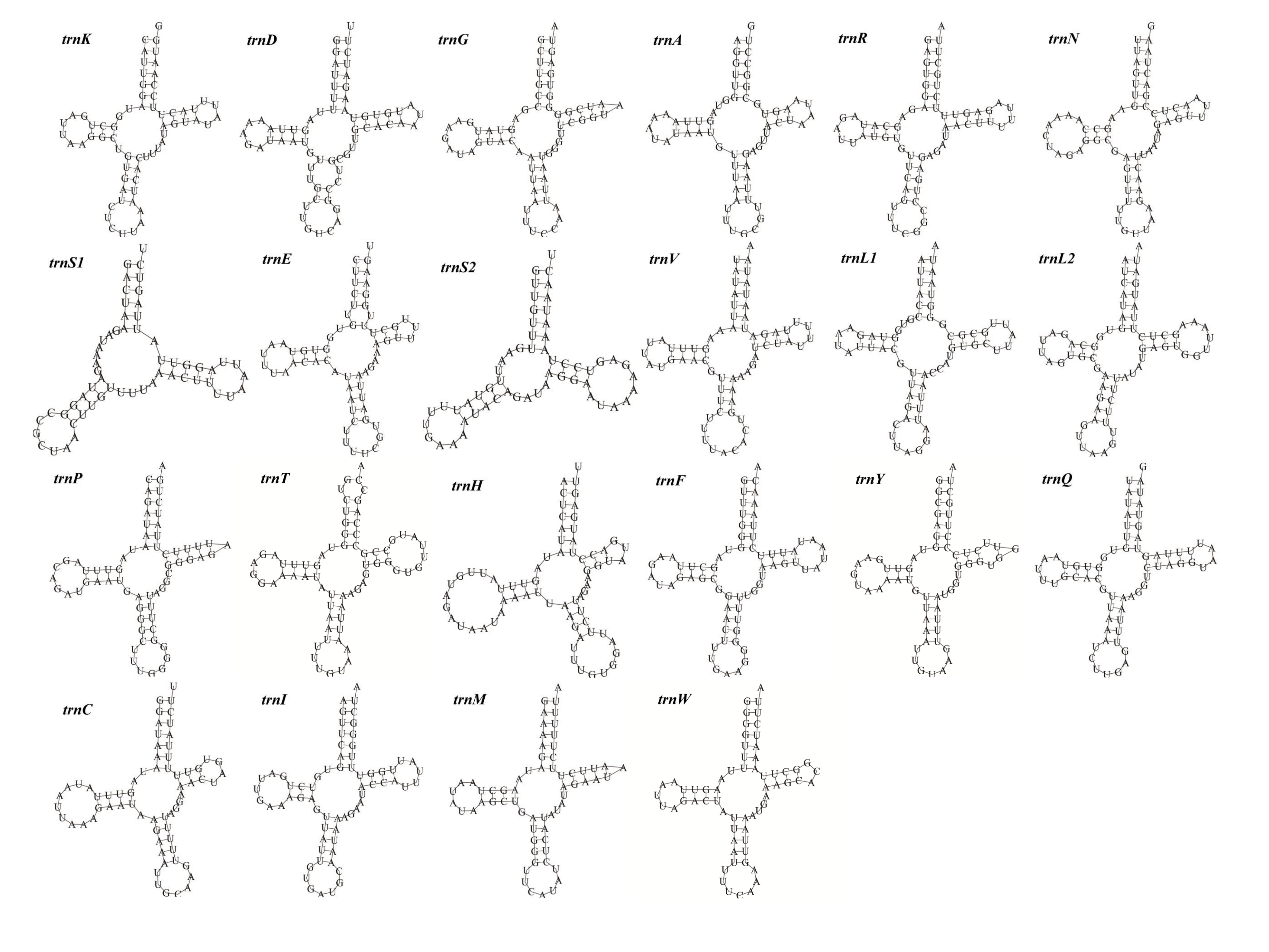


**Figure S2.** Potential secondary structures of 22 inferred tRNAs in *Polydesmus* sp. GZCS-2019.


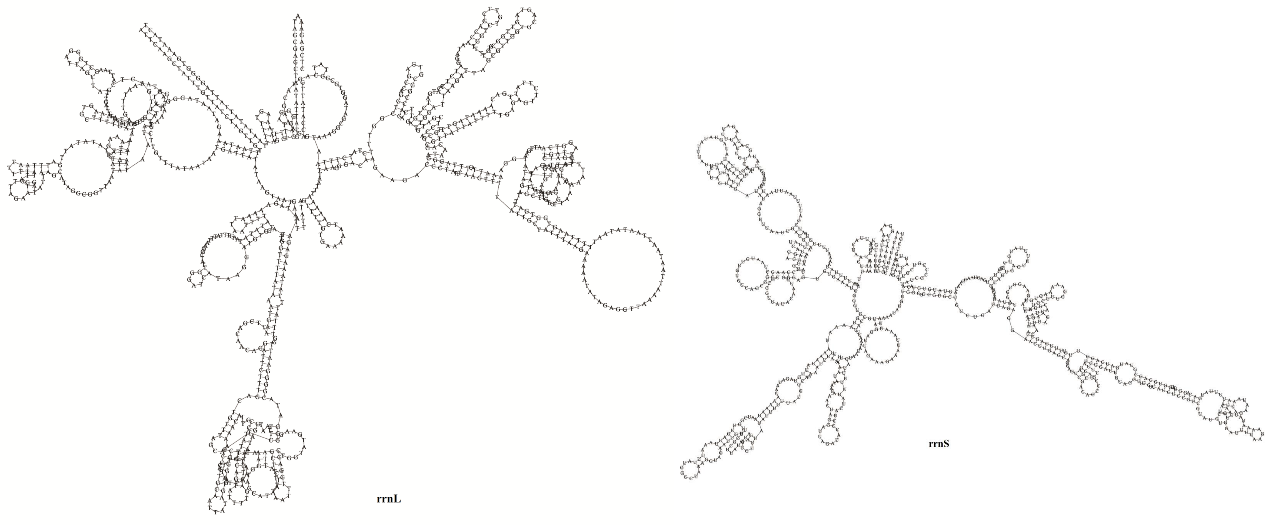


**Figure S3.** Potential secondary structures of two inferred rRNAs in *Polydesmus* sp. GZCS-2019.


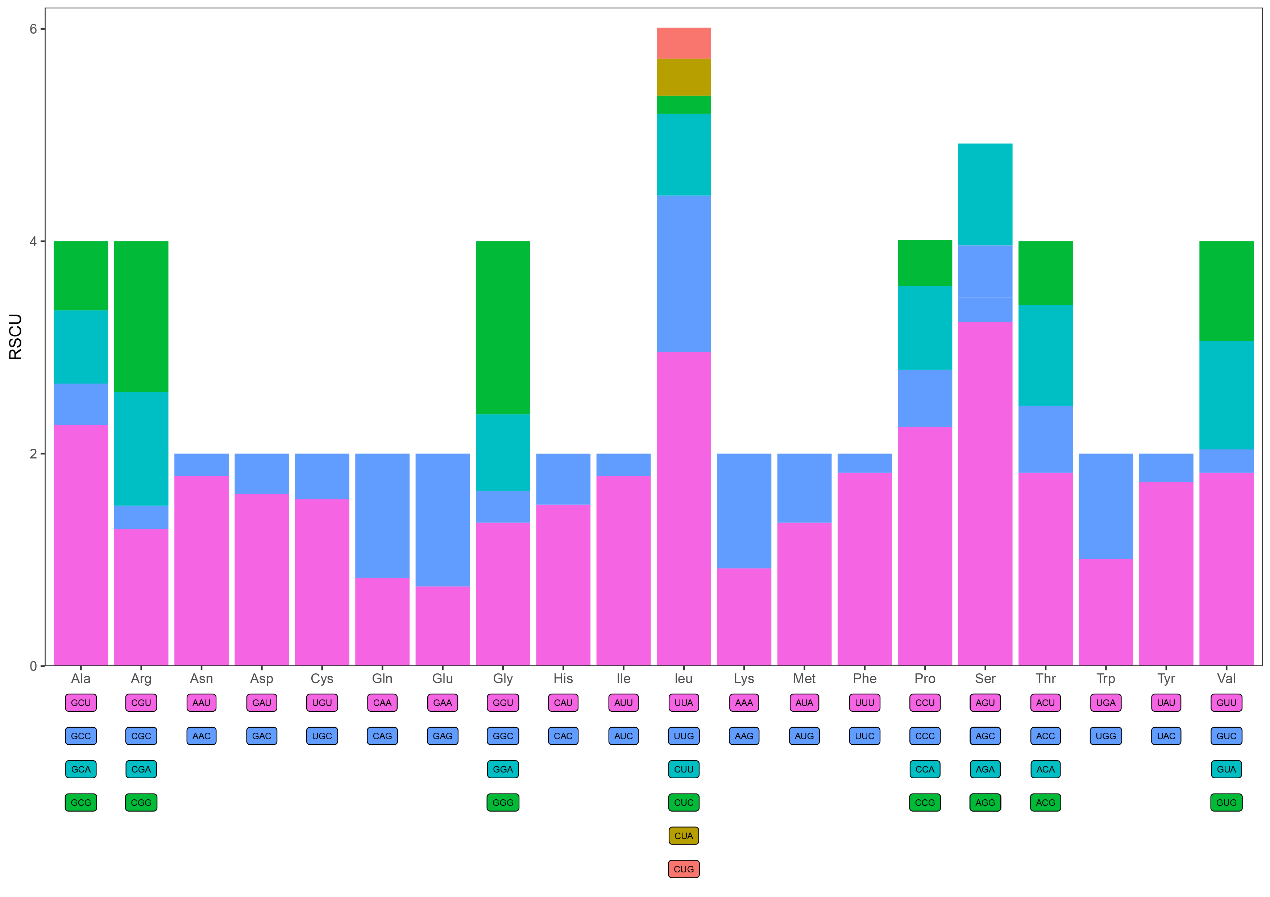


**Figure S4.** Relative synonymous codon usage (RSCU) in the mitogenome of *Polydesmus* sp. GZCS-2019. Codon families are on the x–axis.


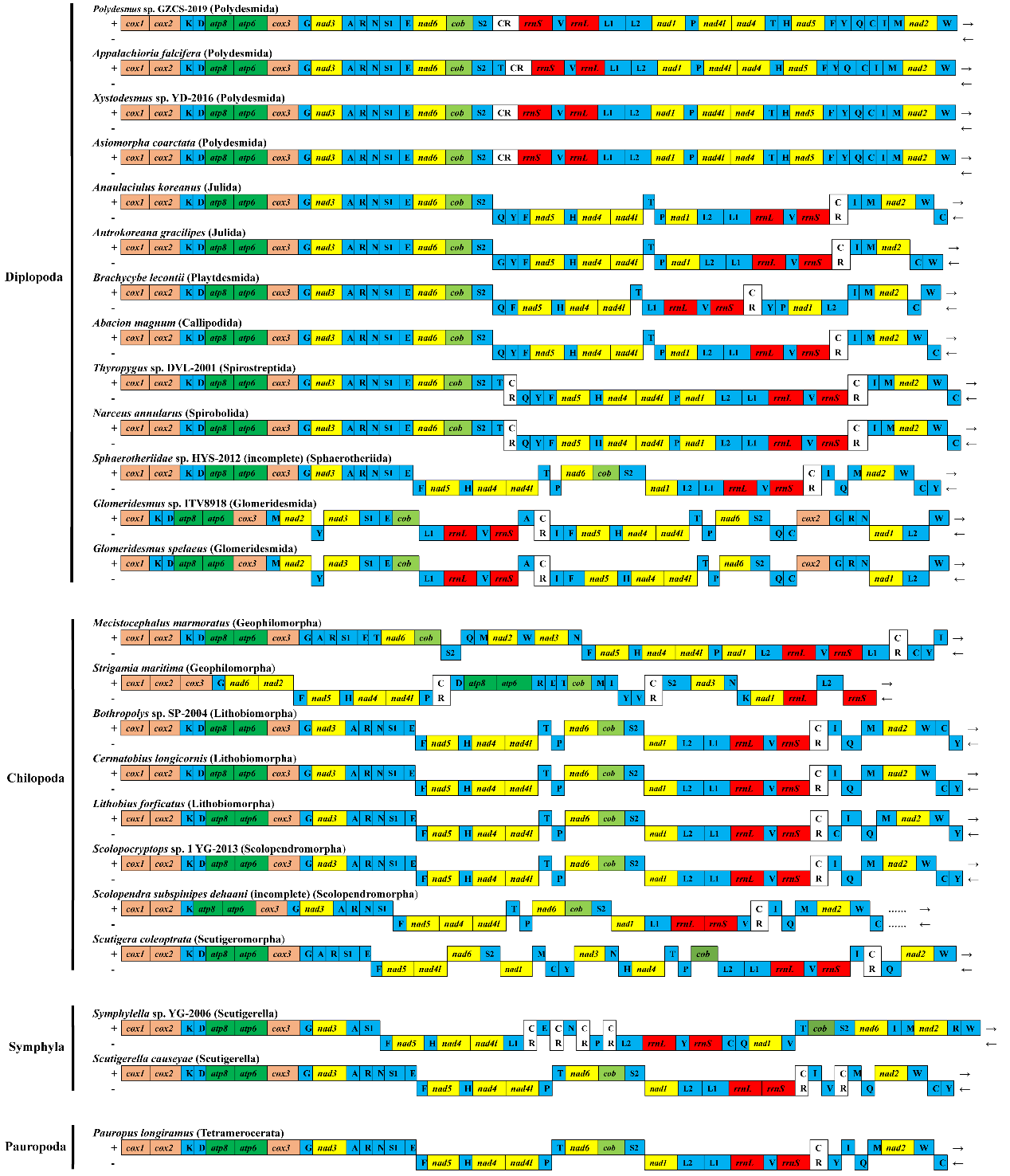


**Figure S5.** Comparison of gene arrangements in mtDNA of the arthropod ground pattern, 13 Diplopoda species, 8 Chilopoda species, 2 Symphyla species, 1 Pauropoda species. Each gene is represented by a specific color. The up and down blocks represent the position on the plus or the minus strand of the genes.


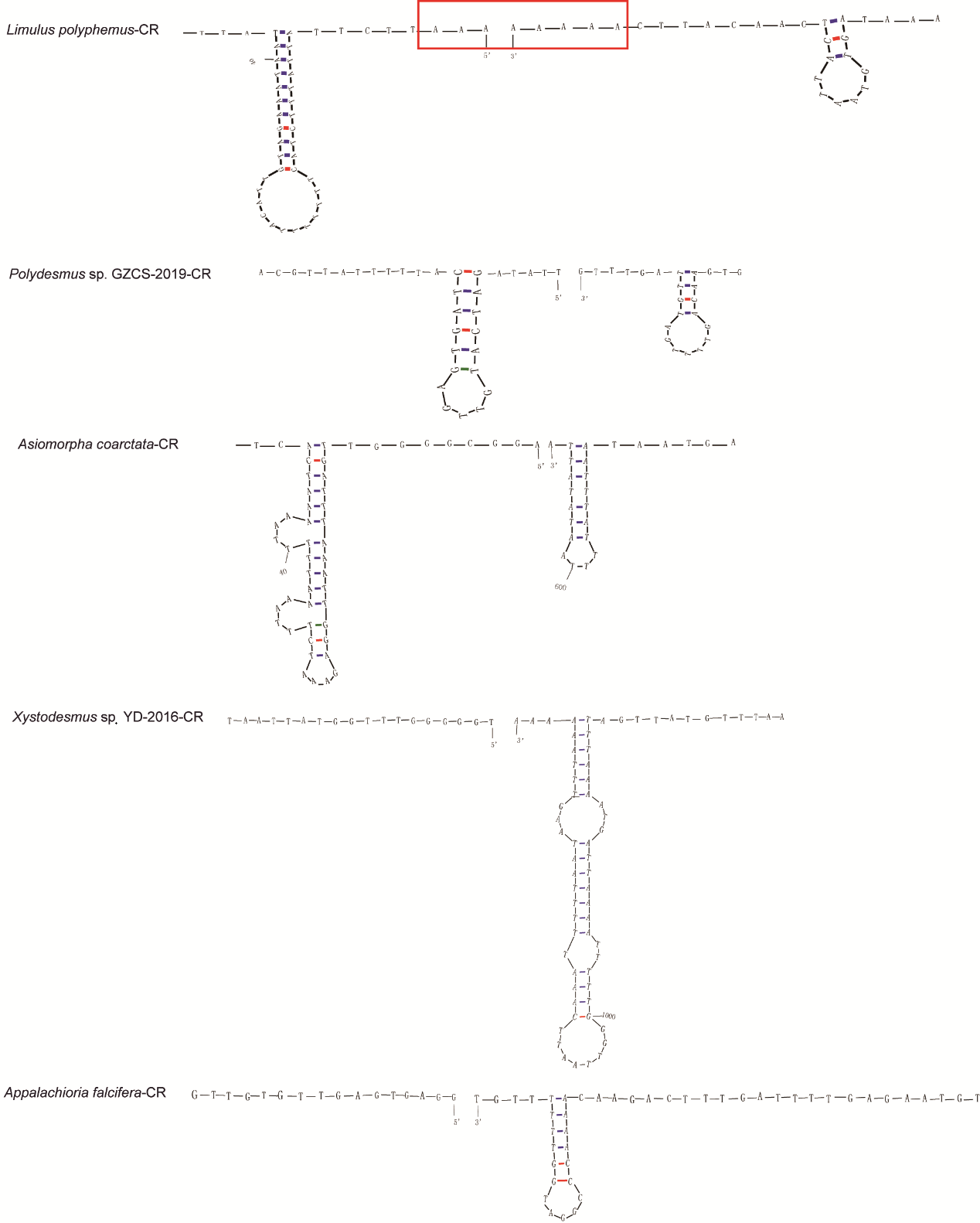


**Figure S6.** Structures of the 5′ and 3′ end of non-coding region in *Limulus polyphemus*, *Polydesmus* sp. GZCS-2019, *Xystodesmus* sp. YD-2016 and *Asiomorpha coarctata*.


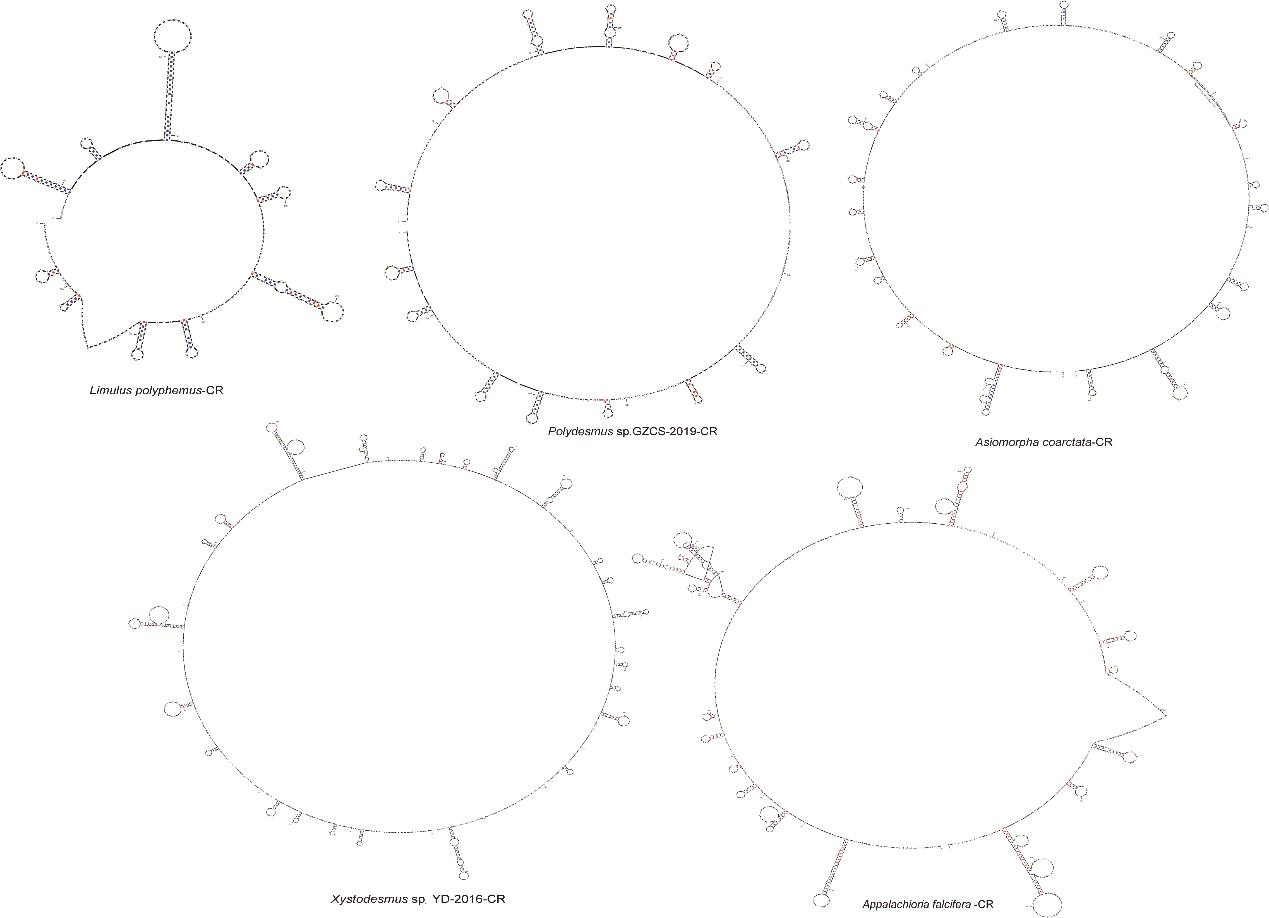


**Figure S7.** Structures of the non-coding region in *Limulus* *Polyphemus*, *Polydesmus* sp. GZCS-2019, *Xystodesmus* sp. YD-2016, *Asiomorpha* *coarctata* and *Appalachioria falcifera*.

**Table S1.** Codon number and relative synonymous codon usage (RSCU) within *Polydesmus* sp.GZCS-2019 mitochondrial genome.

| Codon | Count | RSCU | Codon | Count | RSCU | Codon | Count | RSCU | Codon | Count | RSCU |
| --- | --- | --- | --- | --- | --- | --- | --- | --- | --- | --- | --- |
| UUU(F) | 420 | 1.82 | UCU(S) | 98 | 1.72 | UAU(Y) | 212 | 1.73 | UGU(C) | 102 | 1.57 |
| UUC(F) | 42 | 0.18 | UCC(S) | 13 | 0.23 | UAC(Y) | 33 | 0.27 | UGC(C) | 28 | 0.43 |
| UUA(L) | 319 | 2.96 | UCA(S) | 55 | 0.96 | UAA(*) | 116 | 1.23 | UGA(W) | 106 | 1.01 |
| UUG(L) | 158 | 1.47 | UCG(S) | 18 | 0.32 | UAG(*) | 72 | 0.77 | UGG(W) | 104 | 0.99 |
| CUU(L) | 83 | 0.77 | CCU(P) | 63 | 2.25 | CAU(H) | 54 | 1.52 | CGU(R) | 29 | 1.29 |
| CUC(L) | 18 | 0.17 | CCC(P) | 15 | 0.54 | CAC(H) | 17 | 0.48 | CGC(R) | 5 | 0.22 |
| CUA(L) | 38 | 0.35 | CCA(P) | 22 | 0.79 | CAA(Q) | 25 | 0.83 | CGA(R) | 24 | 1.07 |
| CUG(L) | 31 | 0.29 | CCG(P) | 12 | 0.43 | CAG(Q) | 35 | 1.17 | CGG(R) | 32 | 1.42 |
| AUU(I) | 264 | 1.79 | ACU(T) | 52 | 1.82 | AAU(N) | 145 | 1.79 | AGU(S) | 87 | 1.52 |
| AUC(I) | 31 | 0.21 | ACC(T) | 18 | 0.63 | AAC(N) | 17 | 0.21 | AGC(S) | 28 | 0.49 |
| AUA(M) | 177 | 1.35 | ACA(T) | 27 | 0.95 | AAA(K) | 75 | 0.92 | AGA(S) | 98 | 1.72 |
| AUG(M) | 86 | 0.65 | ACG(T) | 17 | 0.6 | AAG(K) | 88 | 1.08 | AGG(S) | 60 | 1.05 |
| GUU(V) | 190 | 1.82 | GCU(A) | 98 | 2.27 | GAU(D) | 112 | 1.62 | GGU(G) | 159 | 1.35 |
| GUC(V) | 23 | 0.22 | GCC(A) | 17 | 0.39 | GAC(D) | 26 | 0.38 | GGC(G) | 35 | 0.3 |
| GUA(V) | 106 | 1.02 | GCA(A) | 30 | 0.69 | GAA(E) | 54 | 0.75 | GGA(G) | 85 | 0.72 |
| GUG(V) | 98 | 0.94 | GCG(A) | 28 | 0.65 | GAG(E) | 90 | 1.25 | GGG(G) | 192 | 1.63 |

*: the termination codons.

**Table S2.** Composition and skewness of all mitogenomes in this study.

| **Species** | **Whole genome** | | | | **PCGs** | | **tRNAs** | | **rRNAs** | | **A+T-rich region** | |
| --- | --- | --- | --- | --- | --- | --- | --- | --- | --- | --- | --- | --- |
|  | **Size(bp)** | **AT(%)** | **AT skew** | **GC skew** | **Size(bp)** | **AT(%)** | **Size(bp) /number** | **AT(%)** | **Size(bp)** | **AT(%)** | **Size(bp)** | **AT(%)** |
| ***Polydesmus* sp. GZCS-2019** | **15,036** | **66.1** | **-0.236** | **0.429** | **1,0997** | **64.9** | **1,415/22** | **67.8** | **1,862** | **70.2** | **437** | **73.0** |
| *Appalachioria falcifera* | 15,282 | 64.0 | -0.368 | -0.441 | 11,008 | 63.1 | 1,363/22 | 66.5 | 2,025 | 68.7 | 880 | 60.6 |
| *Xystodesmus* sp. YD-2016 | 15,791 | 67.0 | -0.217 | 0.471 | 11,030 | 65.6 | 1,427/22 | 72.0 | 2,007 | 69.2 | 1,032 | 70.1 |
| *Asiomorpha coarctata* | 15,644 | 67.4 | -0.235 | 0.429 | 11,021 | 66.1 | 1,437/22 | 69.5 | 2,016 | 69.3 | 963 | 75.9 |
| *Anaulaciulus koreanus* | 14,916 | 75.1 | -0.040 | -0.127 | 11,036 | 74.6 | 1,365/22 | 77.8 | 1,924 | 75.4 | 590 | 77.5 |
| *Antrokoreana gracilipes* | 14,747 | 62.1 | -0.041 | -0.049 | 11,079 | 61.0 | 1,408/22 | 67.8 | 2,083 | 65.1 | 225 | 60.9 |
| *Brachycybe lecontii* | 15,115 | 76.7 | 0.024 | -0.320 | 11,012 | 75.3 | 1,406/22 | 79.2 | 2,107 | 82.1 | 366 | 74.6 |
| *Abacion magnum* | 15,160 | 66.6 | 0.102 | -0.429 | 11,001 | 64.7 | 1,410/22 | 71.9 | 2,271 | 70.4 | 438 | 76.9 |
| *Thyropygus* sp. DVL-2001 | 15,133 | 67.8 | 0.077 | -0.295 | 10,998 | 66.2 | 1,397/22 | 72.1 | 2,047 | 72.9 | 220 | 81.8 |
| *Narceus annularus* | 14,868 | 63.7 | 0.069 | -0.397 | 10,980 | 62.2 | 1,370/22 | 67.2 | 2,075 | 68.7 | 427 | 71.4 |
| *Sphaerotheriidae* sp. HYS-2012 | 14,970^#^ | 71.2 | 0.064 | -0.355 | 11,053 | 70.2 | 1,373/22 | 75.6 | 2,091 | 74.0 | 387 | 67.7 |
| *Glomeridesmus* sp. ITV8918 | 14,848 | 76.8 | -0.047 | 0.345 | 10,893 | 76.2 | 1,374/22 | 79.8 | 1,715 | 79.1 | 530 | 68.7 |
| *Glomeridesmus spelaeus* | 14,819 | 76.5 | 0.048 | -0.348 | 10,860 | 76.0 | 1,360/22 | 79.6 | 1,715 | 78.0 | 532 | 69.5 |
| *Mecistocephalus marmoratus* | 15,279 | 69.5 | 0.119 | -0.332 | 11,033 | 67.1 | 1,390/22 | 74.5 | 2,128 | 74.2 | 766 | 83.2 |
| *Strigamia maritima* | 14,983 | 64.0 | 0.217 | -0.329 | 11,028 | 62.1 | 858/15 | 68.8 | 2,111 | 68.6 | 969 | 72.1 |
| *Bothropolys* sp. SP-2004 | 15,139 | 70.6 | 0.068 | -0.311 | 10,983 | 68.6 | 1,336/22 | 75.8 | 1,909 | 72.6 | 978 | 81.8 |
| *Cermatobius longicornis* | 16,833 | 63.4 | 0.095 | -0.317 | 10,984 | 60.6 | 1,409/22 | 69.5 | 2,010 | 65.7 | 1,168 | 72.2 |
| *Lithobius forficatus* | 15,695 | 67.9 | 0.087 | -0.269 | 11,026 | 65.7 | 1,336/22 | 70.7 | 1,951 | 71.6 | 1,540 | 77.0 |
| *Scolopocryptops* sp. 1 YG-2013 | 15,119 | 71.6 | 0.031 | -0.313 | 10,920 | 70.3 | 1,433/22 | 77.4 | 1,976 | 73.7 | 861 | 74.4 |
| *Scolopendra subspinipes dehaani* | 14,538^#^ | 74.1 | 0.130 | -0.337 | 10,879 | 73.4 | 1,070/17 | 76.4 | 1,985 | 75.0 | 732 | 77.9 |
| *Scutigera coleoptrata* | 14,922 | 69.4 | 0.043 | -0.314 | 11,086 | 68.3 | 1,383/22 | 73.2 | 1,958 | 71.2 | 479 | 77.9 |
| *Symphylella* sp. YG-2006 | 14,667 | 72.2 | 0.178 | -0.358 | 10,842 | 69.9 | 1,415/22 | 78.7 | 1,766 | 78.1 | 569 | 81.5 |
| *Scutigerella causeyae* | 14,637 | 72.6 | -0.009 | -0.301 | 10,926 | 71.4 | 1,373/22 | 75.2 | 1,890 | 77.6 | 445 | 74.8 |
| *Pauropus longiramus* | 14,487 | 72.9 | 0.012 | -0.362 | 10,869 | 71.2 | 1,470/22 | 77.7 | 1,729 | 78.3 | 609 | 80.5 |
